# Short and long period growth markers of enamel formation distinguish European Pleistocene hominins

**DOI:** 10.1038/s41598-020-61659-y

**Published:** 2020-03-13

**Authors:** Mario Modesto-Mata, M. Christopher Dean, Rodrigo S. Lacruz, Timothy G. Bromage, Cecilia García-Campos, Marina Martínez de Pinillos, Laura Martín-Francés, María Martinón-Torres, Eudald Carbonell, Juan Luis Arsuaga, José María Bermúdez de Castro

**Affiliations:** 1 Centro Nacional de Investigación sobre la Evolución Humana (CENIEH), Paseo Sierra de Atapuerca 3, 09002 Burgos, Spain; 2 Department of Anthropology, University College London, London, WC1H 0BW UK; 3 Equipo Primeros Pobladores de Extremadura, Casa de Cultura Rodríguez Moñino, Cáceres, Spain; 4 Centre for Human Evolution Research (CHER), Department of Earth Sciences, Natural History Museum, London, SW7 5BD UK; 5 Department of Cell and Developmental Biology, University College London, Gower Street, London, WC1E 6BT UK; 6 Department of Basic Science and Craniofacial Biology, New York University College of Dentistry, New York, USA; 7 University of Bordeaux, CNRS, MCC, PACE, UMR 5199 F_33615, Pessac, Cedex France; 8 Institut Català de Paleoecologia Humana i Evolució Social (IPHES), Zona Educacional 4, Campus Sescelades, Edifici W3, Universitat Rovira i Virgili, Tarragona, Spain; 9 Àrea de Prehistòria, Universitat Rovira i Virgili, Avinguda de Catalunya 35, 43002 Tarragona, Spain; 10 Centro mixto UCM-ISCIII de Evolución y Comportamiento humanos, Madrid, Spain; 11 Departamento de Geodinámica, Estratigrafía y Paleontología, Facultad de Ciencias Geológicas, Universidad Complutense de Madrid, Madrid, Spain

**Keywords:** Biological anthropology, Developmental biology, Enamel

## Abstract

Characterizing dental development in fossil hominins is important for distinguishing between them and for establishing where and when the slow overall growth and development of modern humans appeared. Dental development of australopiths and early *Homo* was faster than modern humans. The Atapuerca fossils (Spain) fill a barely known gap in human evolution, spanning ~1.2 to ~0.4 million years (Ma), during which *H. sapiens* and Neandertal dental growth characteristics may have developed. We report here perikymata counts, perikymata distributions and periodicities of all teeth belonging to the TE9 level of Sima del Elefante, level TD6.2 of Gran Dolina (*H. antecessor*) and Sima de los Huesos. We found some components of dental growth in the Atapuerca fossils resembled more recent *H. sapiens*. Mosaic evolution of perikymata counts and distribution generate three distinct clusters: *H. antecessor*, Sima de los Huesos and *H. sapiens*.

## Introduction

Dental enamel microstructure provides a timescale for dental development in the past. It also has the power to resolve taxonomic attribution when morphological character complexes and the timing of tooth formation are combined. Studies on dental enamel growth and relative dental development have been carried out in different hominin populations, from australopiths^1–4^, to early *Homo*^5^, *H. naledi*^6^, a Chinese specimen of the early Late Pleistocene^7,8^, Neandertals^9–12^, and archaic/modern *H. sapiens*^13–16^. However, some of these works focused exclusively on the anterior dentition^1,2,5,6,10^ while others employed crude estimates to reconstruct missing portions of the worn crown^17^. These studies suggest that australopiths and early *Homo* shared dental development that was faster than in modern humans, with suggestions that the shift toward fully modern human dental development appeared some time after *H. erectus* or relatively late in human evolution^5^. This potential shift was observed from a relatively reduced number of teeth, as KNM-ER 15000 and Sangiran S7-37. However, caution is claimed because substantial intraspecific variation in aspects of dental development (such as long-period line periodicity) within early hominin taxa has subsequently been demonstrated^4^. Later Neandertals appear not to have an identical dental development with *H. sapiens*, as some aspects of their dental development tend to be more advanced^9,10,12^. However, a recent study of a single Neandertal from El Sidrón (Spain) suggests that this hominin, with some exceptions, was encompassed in its development within modern human variation^11^. Thus, the key features that characterize *H. sapiens* and Neandertal dental development (and brain size) may have developed separately in parallel in one or more hominin groups during the Middle-Upper Pleistocene.

Three paleontological sites of the Atapuerca complex (Burgos, Spain) fill this potentially crucial and barely known temporal gap in the fossil record of human evolution, although their phylogenetic relationships with later Neandertals and modern humans are intensively debated. The level TE9 of the Sima del Elefante site has yielded a mandible with teeth and a manual phalanx of an undetermined hominin dated to 1.3-1.1 Ma^18–20^. In unit TD6.2 of the younger Gran Dolina site (~0.9-0.8 Ma), more than 160 human fossils representing at least 8 individuals attributed to the species *H. antecessor* have been recovered^21–24^. The more recent Sima de los Huesos site (~0.43 Ma), contains more than 7000 human fossils ascribed to at least 28 individuals whose taxonomical attribution is still under discussion, although genetic and morphological data strongly suggest that the Sima de los Huesos hominins are likely Neandertal ancestors^25^. The phylogenetic relationships among these three archaeological populations are still unknown^18,19,21,22,25^.

Dental development can be studied from two complementary approaches: absolute timing of tooth formation and relative timing, or pattern, of dental maturation. The data relating to the absolute timing of lateral enamel formation are presented here. Perikymata are long-period growth increments that appear on the enamel surface of teeth. They are often easily seen on fossil teeth and are associated with regular underlying long-period growth increments within the enamel called striae of Retzius. Cross-striations within enamel are finer short-period increments of growth visible along enamel prisms that run between and across the long-period striae of Retzius. They are produced by a 24-hour circadian rhythm in enamel matrix secretion. The number of short-period cross-striations, and therefore days, between adjacent long-period striae (of Retzius) defines the periodicity of the perikymata and long-period striae. In hominins this is commonly between ~7–10 days, although more extreme values have been reported^4^. When compared to modern humans, previous studies on perikymata number expressed on the anterior dentition in *H. antecessor* and Sima de los Huesos hominins, showed that Atapuerca Early and Middle Pleistocene hominins had a lower number of perikymata than *H. sapiens*^10,26^. However, some caution is called for since previous studies on these hominins lack direct observations of short-period (daily) enamel growth increments (cross-striations) in individuals and the numbers observed between adjacent long-period striae of Retzius (periodicity). Moreover, the methods used to reconstruct worn teeth in previous studies are imprecise and in many studies the posterior dentition (premolars and molars) were not included in their analyses.

To address the possibility that the Atapuerca hominins share a suite, or pattern, of dental developmental characteristics with *H. sapiens*, we analyzed key aspects of enamel development including lateral enamel growth patterns of all tooth types. Lateral enamel formation time was estimated by combining observations and measurements of daily cross striations, long-period striae of Retzius and the individual periodicities^27^ across the whole dentition in as many of these Atapuerca hominin samples as possible spanning ~1.3 to 0.43 Ma. We also developed a new methodology to reconstruct minimally worn teeth.

## Results

Perikymata number, perikymata distribution and periodicity are useful tools with which to address patterns of enamel and crown development. We evaluated the number and distribution of perikymata over 286 teeth: 96 from Sima de los Huesos, 22 from *H. antecessor* and 168 unworn teeth from a sample of different *H. sapiens* populations (Table S1). Some of these fossil teeth were slightly worn, so their original crown heights were estimated by employing a new method of reconstruction (Supplementary Text 1). Finally, we calculated the periodicities of 14 naturally broken teeth from from hominins of the TE9 level of Sima del Elefante, TD6.2 of Gran Dolina, and Sima de los Huesos.

### Total perikymata counts

It was only possible to assess total perikymata counts along complete unworn crowns in *H. sapiens* and some Sima de los Huesos teeth, but unfortunately this was not possible in any teeth of *H. antecessor* (Fig. S1). Neither was it possible to compare incisors and first molars from Sima de los Huesos as all were worn or did not preserve every perikyma on the enamel surface.

The total perikymata number of some Sima de los Huesos teeth clearly fall below and beyond the range of variation in *H. sapiens*, whereas others are placed in the lower tail of their distribution. Upper and lower M2s and M3s, and lower P4s also have perikymata counts below the range of variation of *H. sapiens*. The interquartile ranges of perikymata counts in samples of upper P3 and lower C do not overlap, and mean counts for *H. sapiens* are always greater than Sima de los Huesos. However, their ranges of variation partially overlap. Finally, and in contrast to other tooth types, in upper Cs and in upper P4s and lower P3s the interquartile ranges of the Sima de los Huesos sample entirely overlap with *H. sapiens*.

### Patterns of perikymata distribution

**Figure 1:**
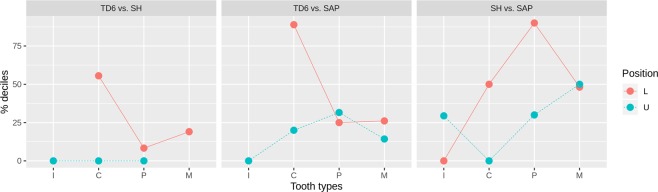
Percentage of deciles that do not overlap their 95% confidence limits of the regression equations formed to summarize the perikymata distribution along the crown heights. Sima de los Huesos (SH), Gran Dolina-TD6 (TD6) and *H. sapiens* (SAP). Lower and upper dentitions (L and U, respectively) in incisors (I), canines (C), premolars (P) and molars (M).

**Figure 2:**
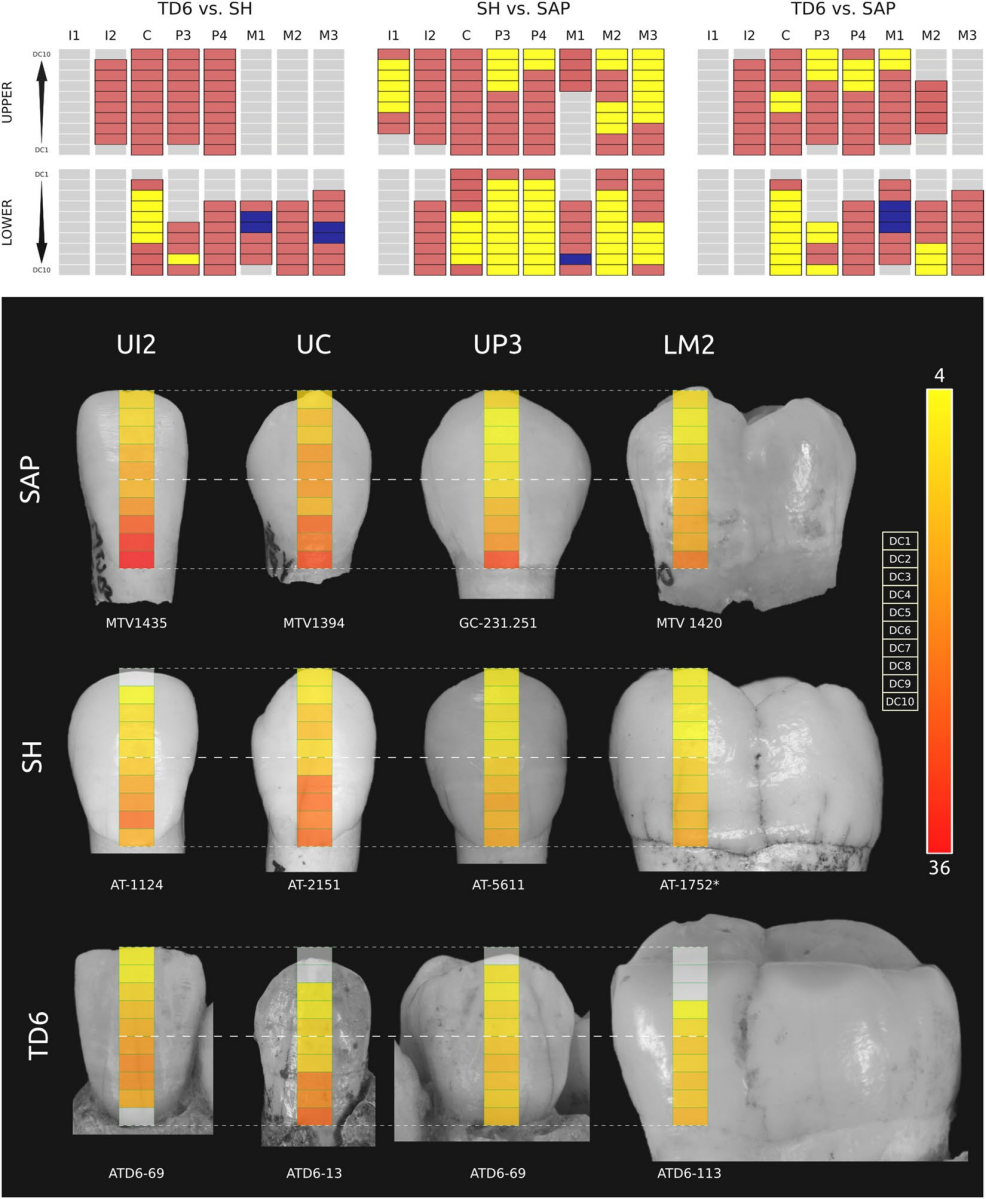
Perikymata distribution along crown heights. Sima de los Huesos (SH), Gran Dolina-TD6 (TD6) and *H. sapiens* (SAP). (**a**) Color code representing if 95% confidence limits of two populations overlap or not per decile and tooth. Red: overlap; yellow: no overlap, the second population contains more perikymata than the first population; dark blue: no overlap, the second population contains less perikymata than the first population. (**b**) Number of perikymata per decile of crown height (from deciles 1 to 10): upper lateral incisor (UI2), upper canines (UC), upper third premolars (UP3) and lower second molars (LM2). Note that AT-1124, ATD6-13, ATD6-69 UP3 and ATD6-113 crown heights have been reconstructed using the statistical method described in the main text. Blank deciles mean that perikymata counts could not be made. (*) this image has been horizontally flipped. Tooth crowns are shown at equal crown height, not to scale. Scale bar: number of perikymata, from 4 (yellow) to 36 (red).

Perikymata distribution along the crown heights, measured as the number of perikymata per decile of height, define a pattern. It seems that this pattern evolved asynchronously when all tooth types are considered (Figs. 1 and 2). These results point to a different rate of development for each stage or decile of each permanent tooth type (incisors, canines, premolars and molars).

Considering the anterior dentition (Fig. 1), the upper incisors in *H. antecessor* do not show differences with later populations including Sima de los Huesos and *H. sapiens*. Lower incisors from Sima de los Huesos hominins do not show differences with later populations either.

Comparing the canines of *H. antecessor* with those of all later populations, a different pattern emerges concerning their position (Fig. 1). Lower canines tend to have a remarkably higher percentage of deciles that do not overlap with other populations compared to the percentages displayed in upper canines, which indicates that the perikymata distribution of upper canines is more similar to later populations than the distribution observed in lower canines. In this regard, upper canines show no differences when *H. antecessor* and Sima de los Huesos are compared, whereas these differences increase between ~12 and ~23% when they are compared with *H. sapiens*. The percentages of difference regarding their position increased up to ~50% when Sima de los Huesos and *H. sapiens* are compared. Remarkably, there seems to be no difference in upper canine perikymata distribution when Sima de los Huesos and *H. sapiens* are compared.

Regarding patterns of perikymata distribution in premolars (Fig. 1), *H. antecessor* and Sima de los Huesos present practically identical patterns with very low differences between upper and lower premolars. However, when *H. antecessor* is compared with the *H. sapiens* sample, about 25% of deciles differ, although no differences are observable between upper and lower premolars. Differences between upper and lower premolars emerge when Sima de los Huesos and *H. sapiens* are compared. When these samples are compared, the difference between upper and lower premolars reach values of ~60% in favor of lower premolars.

*H. antecessor* lower molars have ~20–35% of deciles which do not overlap with later populations (Fig. 1). When Sima de los Huesos and *H. sapiens* are compared among them, lower molars tend to have between 40 and 60% of differences. In this case, we cannot evaluate differences among upper and lower molar patterns in all comparisons. In those cases, where both molars are preserved, as it happens when *H. antecessor* and Sima de los Huesos are compared with *H. sapiens*, these differences are very low, from ~0 to ~10%.

These results suggest that the lower and upper dentition, as well as some groups of teeth, changed over time following different growth rates. This possible pattern of mosaic evolution indicates that lower teeth could have evolved more rapidly than upper teeth in this respect. Incisors and first molars were relatively stable whereas lower canines underwent the most significant shifts.

### Difference in perikymata number

**Figure 3:**
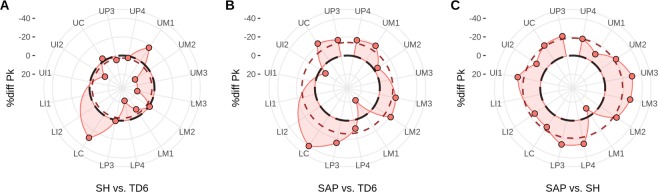
Mean percentage of difference in number of perikymata in all the teeth between Sima de los Huesos (SH) and *H. antecessor* (TD6) (**A**), *H. sapiens* (SAP) and TD6 (**B**), and SAP and SH (**C**). In all plots the reference species is first in the comparison and is represented by a black long-dashed circle that equals 0. Differences situated outwards the reference species indicate teeth present fewer perikymata, and those inwards the circle have more perikymata. The mean percentage of difference considering all teeth together is represented by a colored short-dashed line. Because not all fossil teeth were unworn or preserved, the percents were calculated by summing the perikymata only in the deciles available on both species (see also see Figs. S4 and S5).

The difference in total perikymata counts per tooth type and the average difference for the pooled sample of all teeth, both measured as percentages, can be seen in Fig. 3. These differences were calculated by comparing the number of perikymata only in the deciles where the two populations considered do have a known number of perikymata.

Sima de los Huesos hominins present on average a perikymata count that is 18.79% less than *H. sapiens* when all teeth are considered together, while this difference falls to 14.05% less perikymata compared to *H. antecessor*. When both fossil samples are compared, we see that *H. antecessor* has 2.75% more perikymata than Sima de los Huesos.

The difference between the patterns of perikymata between *H. sapiens* and the sample from the Sima de los Huesos is similar for all types of teeth (Fig. 3C). Most of the teeth of the *H. sapiens* sample exhibit approximately 20% more perikymata than the teeth of the Sima de los Huesos, with the exception of some molars. Thus, the lower M1 of Sima de los Huesos presents 2.75% more perikymata than its counterpart in the sample of *H. sapiens*.

Comparing *H. antecessor* with the other samples, we see that lower canines are particularly interesting (Fig. 3A, B). Lower canines present on average a difference between 20% to 40% less perikymata than Sima de los Huesos (~30%) and *H. sapiens* (~40%). Incisors are only represented by the upper I2, which is very close in all the species with the average value of *H. antecessor* (ranging from 0 to 10%). The teeth of *H. sapiens* tend to have more perikymata, or are close to the average of *H. antecessor*. The exception is the lower M1, which has less perikymata than *H. sapiens*.

### Difference in perikymata number per decile

When mean perikymata counts across deciles between Sima de los Huesos and *H. sapiens* are compared (Fig. S2), two groups of teeth can be identified: a first group containing Sima de los Huesos teeth where the perikymata number in all deciles are below *H. sapiens*, and a second group where one or more Sima de los Huesos deciles present more perikymata than *H. sapiens*. The first group is composed by upper I1, upper C, all premolars except upper P3, lower M2 and upper M3. In this case, these differences can reach values as low as −40%, and they are preferentially located in the deciles 9 and 10. The second group is composed by upper I2 and both first molars (M1), which tend to have more perikymata on average in the deciles deciles 7 to 9 than *H. sapiens*. Lower I2 present more perikymata from deciles 5 to 7. Only four teeth present one decile with more perikymata than *H. sapiens*: lower C and upper P3 (decile 3), upper M2 (decile 7) and lower M3 (decile 6). In all cases in this second group, the higher perikymata values in Sima de los Huesos fall slightly above the average of *H. sapiens*, with the exception of lower M1, which is the only tooth type that contains a higher number of perikymata in respect to *H. sapiens* from decile 8 to 10.

When *H. antecessor* and *H. sapiens* are compared (Fig. S2), two groups can also be distinguished. A first group, characterized by *H. antecessor* presenting a tendency of having more perikymata in at least one decile from deciles 3 to 7 than the *H. sapiens* average, can be observed in lower P4, M1, M3 and upper M2. For instance, these differences can reach values as large as +90% in decile 4 of lower M1. A second group, characterized by teeth with *H. antecessor* perikymata number per decile below or equal than *H. sapiens*, is formed by upper and lower canines and P3, and by upper I2, P4 and M1. Interestingly, none of these *H. antecessor* teeth have more perikymata than *H. sapiens* in the last two deciles (9 and 10).

Four groups of teeth can be identified when *H. antecessor* is compared with Sima de los Huesos hominins (Fig. S2). On the one hand, *H. antecessor* tends to have more perikymata in the cuspal deciles (from 1 to 5) than Sima de los Huesos hominins in all upper teeth with the exception of the P3 and M1. In the case of the upper M1, no data were recorded for the perikymata in cuspal deciles; however, their deciles 6 and 7 have more perikymata than Sima de los Huesos. On the other hand, lower teeth tend to have more perikymata in the cervical deciles (from 6 to 10) in *H. antecessor* than in Sima de los Huesos hominins, with the exceptions of the lower C and lower P4. Each of these two teeth might define one group each. Whereas *H. antecessor* has a lower number of perikymata in all the deciles of the lower C than Sima de los Huesos, lower P4 of *H. antecessor* has more perikymata in all the deciles than the Middle Pleistocene population.

As the majority of the teeth from the Atapuerca hominins were slightly worn, only perikymata numbers present from deciles 6 to 10 were used to run a principal component analysis. The rationale for including the cervical half deciles include: 1) to maximize sample sizes, as slightly worn teeth can be included, 2) to take into consideration the deciles which contain more perikymata, and 3) to evaluate which deciles are more diagnostic to differentiate populations.

The first two principal components (PC1 and PC2) express more than 90% of the variation in five teeth (upper I1, upper and lower M1s, upper M2 and upper M3), two teeth are between 70% and 80% (upper P4 and lower M2), whereas the rest are placed between 80% and 90% (Table S2). Therefore, all these two first principal components contain more than 70% of variation for every tooth type. Loading factors per tooth type in each principal component are shown in Table S3. Deciles 9 and 10, and to a lesser extent decile 8, are the most influencing deciles in PC1.

Principal component analysis’ bivariate plots of all teeth representing PC1 and PCA2 are plotted in Fig. S3. *H. antecessor* tends to be encompassed within Sima de los Huesos variation. Compared with *H. sapiens*, Sima de los Huesos partly overlaps modern human variation, but also expands beyond the *H. sapiens* range of variation by defining their own areas.

### Periodicity

**Figure 4:**
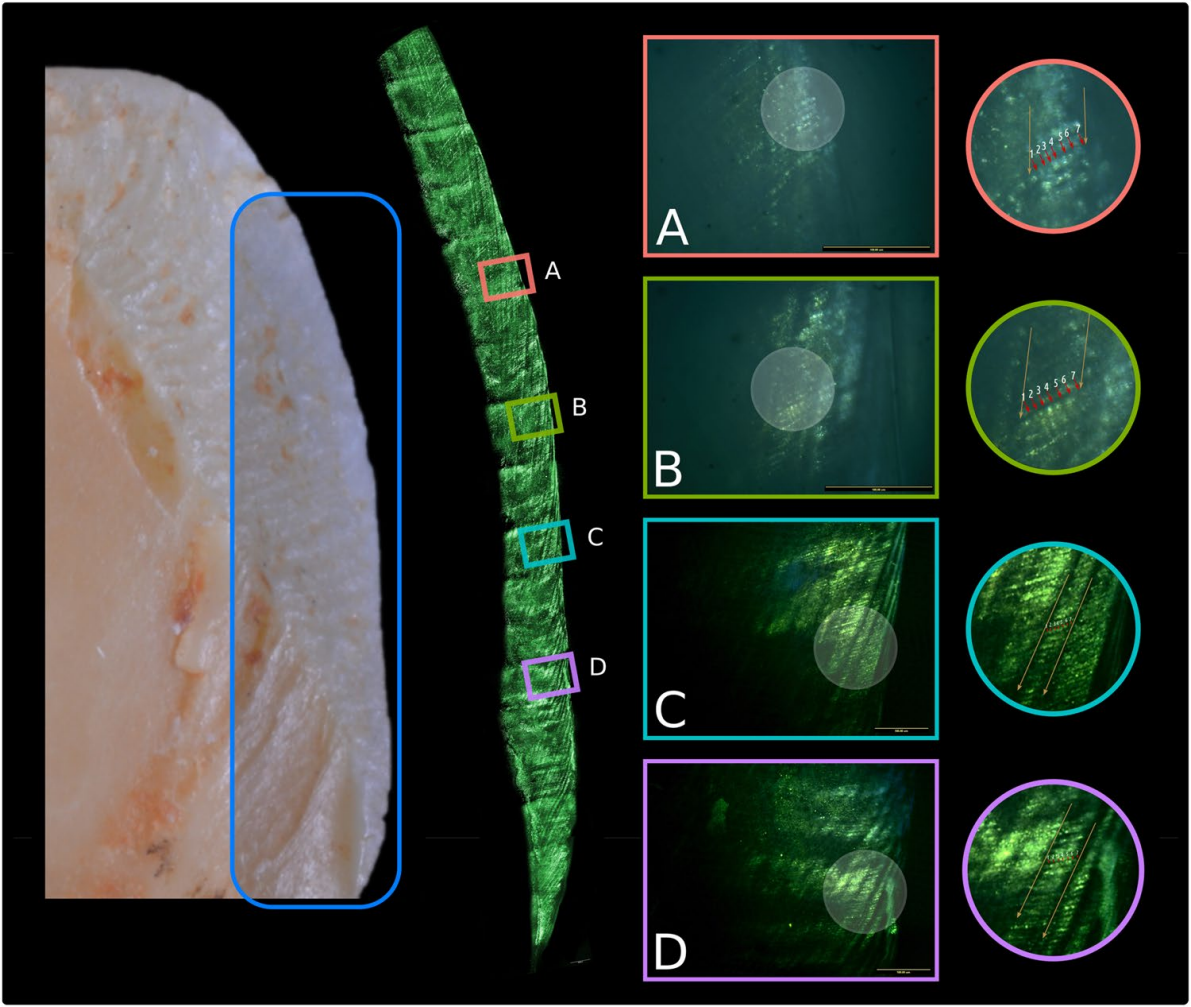
7 days-periodicity of the *H. antecessor* molar ATD6-92. Portable confocal scanning optical microscope was employed to get the images. Circle figures represent Retzius striae (yellow) and cross-striations (red).

Periodicities and daily secretion rates were calculated in fourteen naturally fractured teeth from Sima del Elefante, *H. antecessor* and Sima de los Huesos hominins (Table 1, Fig. 4 and Supplementary Text 2). Four hominins from the Early Pleistocene Sima del Elefante and Gran Dolina-TD6 sites (n = 4, MNI = 4) display periodicities that range from 6 to 7 days (mode = 7), whereas six hominins from the Middle Pleistocene Sima de los Huesos site (n = 10, MNI = 6) display periodicities that range from 6 to 9 days (mode = 7). Out of the 14 periodicity values, 11 are seven days or less, only 3 teeth have periodicity values higher than seven days.

**Table 1:** Cross-striation periodicities between striae of Retzius (Periodicity, days) and daily secretions rates (DSR, µm/day) of the Atapuerca naturally fractured teeth.

Site	Specimen	Tooth	Position	Side	Hominin	Periodicity	ARL	DSR	Enamel region
SH	AT-43	M1	L	L	II	9	5	4.49 ± 0.14 (8)	lat.out
SH	AT-64	P3	L	L	VII	8	4	7.50 ± 0.89 (5)	lat.out
SH	AT-13	M3	L	L	VII	8	4	5.57 ± 0.25 (7)	lat.out
SH	AT-2135	M3	U	R	XVIII	7	4	4.37 ± 0.41 (5)	lat.mid
SH	AT-827	M2	U	L	XIX	6	4	4.72 ± 0.42 (7)	lat.out
SH	AT-1475	C	U	R	?	7	4	4.25 ± 0.39 (14)	cer.out
SH	AT-1942	C	U	L	?	6	4	5.00 ± 0.36 (7)	cer.out
SH	AT-3192	C	U	R	?	6	4	4.22 ± 0.25 (9)	lat.out
SH	AT-6873				?	7	5	3.96 ± 0.31 (6)	lat.out
SH	AT-6874				?	7	4	4.87 ± 0.40 (10)	cer.out
TD6	ATD6-104	dm			?	6	4	2.72 ± 0.31 (15)	lat.mid
TD6	ATD6-6	C	L	R	H1	7	5	5.24 ± 0.52 (4)	lat.out
TD6	ATD6-92	M			?	7	5	4.29 ± 0.35 (5)	lat.out
TE	TE-1	P3	L	L	1	7	5	6.45 ± 0.34 (17)	lat.out

Sites are Sima de los Huesos (SH), Gran Dolina-TD6 (TD6) and Sima del Elefante-TE9 (TE). Position is lower (L) and upper (U). Side is left (L) and rigth (R). DSR shows the average, standard deviation and number of observations. Enamel region represents the area where the DSRs were measured, lateral outer (lat.out), lateral middle (lat.mid), cervical outer (cer.out). ARL shows the number of adjacent Retzius lines employed to calculate DSRs in each tooth; expressed in days it would be ARL - 1. Minimum number of individuals of SH is six, whereas teeth from TD6 and TE correspond to one different individual each.

## Discussion

The Atapuerca hominins fill a time gap of roughly 1 Ma, which is fundamental to discerning how incremental markers of dental development in hominins evolved and how modern humans and Neandertals might have acquired their characteristic dental development and their relatively slow growth pattern. Our data support a scenario of mosaic evolution in perikymata counts and distribution, where each population presents its own taxonomic signal. In this case, modern humans form a distinct group with most of their features not shared with the samples of the European Early-Middle Pleistocene hominins here analyzed.

We observed that the lower dentition shifted from the Early Pleistocene to the Middle Pleistocene more rapidly than the upper dentition in respect to perikymata number and perikymata distribution. These differences in perikymata counts and perikymata packing patterns might result from the mixed developmental origin of the permanent dentition. The morphogenetic growth sites of anterior and posterior teeth are controlled by different homeodomains, and incisors, canines and premolars are formed from successional laminae, whereas molars develop from distal extensions of the primary dental lamina^28^. However, premolars and molars tend to evolve as a single unit^29^, which appears to be the case for *H. antecessor* and Sima de los Huesos. Morphogenesis of the upper versus lower dentition dichotomy is under the control of two different genetic programs^28^. The morphological variation in the lower dentition in Plio-Pleistocene hominins is more extreme than in the upper dentition. These differences in the genetic regulation of the homeodomains of different tooth families, and their mixed developmental origins may allow natural selection to affect tooth types individually.

The difference in the number of perikymata per decile across all teeth, expressed as a percentage, show a similar pattern of mosaic evolution as does perikymata distribution. *H. antecessor* upper incisors do not show a significantly different number of perikymata relative to later populations. This is also the case for the upper canines but not for the lower canines, which are highly modified in Sima de los Huesos and *H. sapiens*. The postcanine dentition of Sima de los Huesos and *H. sapiens* is also different compared to *H. antecessor*, although considerable differences exist between upper and lower teeth. In premolars, only lower P4s of Sima de los Huesos increase ~20% the number of perikymata in respect to *H. antecessor*, while *H. sapiens* premolars are different with respect to all of them. Concerning molars, each one seems to have followed their own independent pace of change.

Modern humans present modal periodicities ranging from 8 to 10 days^14,15,30^. However, lower and higher values in individuals have also been documented (6 and 12 days^30^), although these extreme values are highly residual. Fossil *H. sapiens* present periodicities that overlap with modern humans, as evidenced by a 10-day periodicity of Jebel Irhoud 3^16^, 8-day periodicity in Skhul II and Qafzeh 10^12^, and 7-day periodicity in Hofmeyr and Qafzeh 15^12^. Although limited by sample size, our analysis shows a high level of overlap. Periodicity modal values over time in Europe from Sima del Elefante-TE9 and Gran Dolina-TD6 to Sima de los Huesos and Neandertals^12^ remained unalterable (7 days). For their part, fossil *H. sapiens* (8 days)^12^ and modern humans (between 8 and 9 days) point to an increasing in periodicity modal values with respect to other Pleistocene populations.

Lower periodicity modal values combined with a reduced number of perikymata indicate that enamel formation time in *H. antecessor* and Sima de los Huesos was more rapid than in *H. sapiens*. On average, *H. sapiens* contains 11.30% and 16.18% more perikymata and 15.63% and 10.38% higher periodicity values than *H. antecessor* and Sima de los Huesos respectively, considering 8 days as the *H. sapiens* periodicity average. Remarkably, *H. antecessor* contains on average, 2.75% more perikymata, and 6.22% lower periodicity than Sima de los Huesos. Despite some individual outlier teeth, such as lower canines and molars, the averages indicate relative stasis in enamel formation times from *H. antecessor* to Sima de los Huesos, although the phylogenetic relationship between the two remains uncertain.

Perikymata number in lower canines shifted markedly in Sima de los Huesos compared to *H. antecessor* and the perikymata number of this tooth changed again in *H. sapiens* with respect to all previous populations. This indicates that there were at least three major steps in the evolution of perikymata number in lower canines, with an increasing number from the most ancient population (*H. antecessor*) to the most modern one (*H. sapiens*), although there might be no phylogenetic relationship among them. The greater number of perikymata in the more recent population contrasts with the crown height reduction in *H. sapiens* with respect to the other fossil populations^10^. Perikymata in *H. sapiens* are more densely packed but largely as a result of longer enamel formation times and a greater number expressed on slightly shorter crown heights. Concerning molars, it is particularly interesting that there is stasis in the number of perikymata of M1s from the Middle Pleistocene to *H. sapiens*, which contrasts with the high variation present in the other molars.

This evidence allows us to differentiate three groups: *H. antecessor*, Sima de los Huesos and *H. sapiens*. The most relevant tooth which presents the highest differences in *H. antecessor* respect to the other hominins is the lower canine, which contains a relatively low number of perikymata compared to later populations. Finally, when *H. sapiens* is compared to the other populations, they present more perikymata with the exception of some molars. These differences are mainly found in the high number of perikymata present in the last three deciles. All of these features can be considered as taxonomically significant, which ultimately is connected with the pattern of mosaic evolution displayed in the number and distribution of the perikymata.

Concerning Neandertals, several studies addressed perikymata counts and distribution along crown heights^9,12,30–37^. However, most of these studies employed untested reconstruction methodologies of worn teeth that cannot be directly compared with our results. Moreover, they estimated the number of perikymata lost in the missing part of the enamel. However, tentatively, and by employing published data, the difference (in percentage) of perikymata counts of all teeth combined between Sima de los Huesos and Neandertals is virtually zero, where the highest differences are present in the molar area. Taking into account that the difference in the average periodicity among both populations is low (3.71%), our results on the anterior dentition would not support the hypothesis that Neandertals had shorter periods of dental growth than the Sima de los Huesos hominins^10^. Concerning perikymata distribution, Neandertals might have presented an extraordinarily rectilinear and gradual distribution, differing markedly with Sima de los Huesos and *H. sapiens*, because the latter two groups tend to present comparatively reduced perikymata counts in the cuspal half and more perikymata in the cervical half than Neandertals.

There is a tight correlation between dental development and some life-history traits, such as brain size, age at first reproduction or lifespan^5,10,12,38–40^. However, some have suggested that dental development, as a life-history related variable, is only weakly linked with life-history variables^41^. Certainly, this correlation is high when dealing with high-rank taxonomical categories (i.e. order Primates), but is less reliable when dealing with low-rank categories (i.e. species)^42^. Interestingly, the specific dental development variable used in the majority of these studies is the age at gingival eruption of the lower M1s. Molar crown formation time in primates^43^ and the interval of Retzius line periodicities^44^ were proposed as alternative proxies for estimation of life history traits. The former was discarded because modern humans, chimpanzees and orangutans^45^ overlap extensively whereas the latter has been found to be positively correlated in primates with body mass and other life history variables, as well as with basal and specific metabolic rates^44^.

Although dental development is commonly integrated with child growth^46^, the specific correlation between dental development and skeletal growth has been questioned^47^. Despite dentally advanced modern humans tending to be skeletally advanced, it seems that both variables are only moderately correlated, so that the advanced dental development observed in fossil hominins might have not been accompanied by a faster skeletal growth^47^. Furthermore, dentally advanced children who are skeletally delayed due to malnutrition may have dental development on time or advanced relative to European populations^48^.

Our data demonstrate distinct differences in perikymata number and packing pattern between the fossil samples from *H. antecessor* and Sima de los Huesos hominins. Based on the sum of the mean percentages of difference in perikymata counts in all teeth and on periodicities, we observe that cusp specific lateral enamel of both *H. antecessor* and Sima de los Huesos hominins grew on average a 27% faster than in *H. sapiens*. However, further evidences from cuspal enamel and root formation times, along with total enamel formation times, are required to have a full picture of the absolute timing of dental maturation.

Interestingly, age at death of the Nariokotome child, a 1.53 Ma hominin from east Africa^49^, was estimated between 7.6 and 8.8 years^50,51^. By this age, this specimen had their M2s fully erupted. In the extremely variable *H. sapiens* population, the chronological age at M2 eruption is about 12 years. For instance, the lower M2 eruption ages in a Greek population was 12.01 ± 1.62 for males and 11.73 ± 1.29 for females^52^; in a Nigerian population these ages are 11.58 ± 1.36 and 11.25 ± 1.16 for males and females, respectively^53^; and in Baka pygmies these ages are 10.76 for males (95% CI: 10.52–11.00) and 9.88 for females (95% CI: 9.49–10.23)^54^. This indicates that Nariokotome M2s erupted earlier than average ages of modern humans, although encompassed in the extreme tail of their variation. Compared to modern human averages, overall faster cusp specific lateral enamel formation times and advanced molar development in both fossil populations from Atapuerca could be possibly considered as indicators of a more rapid ontogenetic development. In other words, our data are not incompatible with dental development among the two fossil populations of Atapuerca being at the more advanced end of the dental development of modern humans.

The shifts in dental development over time should be taken with some caution. Although general patterns may be drawn by employing the averages of the data, hominins display a wide variability in enamel microstructure^4,10,12^ which ultimately would lead to misinterpretations when sample sizes are relatively low. In this research we counted perikymata over 118 teeth and calculated periodicities over 14 teeth from the Early to Middle Pleistocene sites of Atapuerca, which represents comparatively one of the largest sample sizes of this period. Despite the observed variability, overall perikymata counts and perikymata distributions show a mosaic evolution, and allow us to discriminate between European Pleistocene hominins. Lower canines of *H. antecessor* have comparatively fewer perikymata than later populations. *H. sapiens* has densely packed perikymata from decile 8 to 10 that are more highly packed on average than any other earlier population. Three sample clusters can be observed: *H. antecessor*, Sima de los Huesos hominins and *H. sapiens*.

## Materials and Methods

### Materials

#### Perikymata counts

Perikymata counts were analyzed on 286 teeth: 96 from Sima de los Huesos and 22 from *H. antecessor* (Table S4), and 168 teeth from different *H. sapiens* populations, either archaeological or modern (Table S1). Lower and upper tooth sample sizes are similarly represented, with 141 and 145 teeth respectively. All *H. sapiens* teeth were unworn (stage 1), while *H. antecessor* and Sima de los Huesos were unworn or slightly worn (stage 2)^55^. Teeth with wear stages beyond 2 were not included. 55 Sima de los Huesos and 18 *H. antecessor* slightly worn fossil teeth were reconstructed using the previous regression equations in order to estimate the location of the highest point of the crown height (Tables S5 and S6) and divide it into deciles. In lower and upper molars, the selected cusps are protoconids and protocones, respectively, whereas in premolars they are the buccal cusps.

To complete this comparative framework, a relatively large sample of *H. sapiens* teeth was employed. These modern human teeth come from 6 origins: five archaeological caves in the Iberian Peninsula (Maltravieso, Santa Ana, El Mirador, Galls Carboners and Guineu) and one from a current Sudanese population. None of these teeth displayed any evidence of wear (category of wear stage 1)^55^.

Maltravieso Cave is located in Cáceres (Extremadura, Spain). This cave was discovered in 1951 in a limestone quarry that affected a room that was named as *Sala del Descubrimiento* (Discovery Room). A thousand ceramic and human remains that were part of a collective grave, and ascribed to belong to the half of the second millennium BC, were uncovered^56–58^.

Santa Ana Cave presents several stratigraphical units that correspond to the Pleistocene^59^. All the remains from the Pleistocene sediments were uncovered in a calcified breccia. However, sediments from historical ages have also been found. Although the exact historical period of the molar remains unknown, its attribution to *Homo sapiens* is unquestioned.

Galls Carboners Cave is located in the Prades Mountains (Tarragona, Spain) and it contains a collective burial was excavated in different periods, and the dating of a human remain places this site in 3,310 ± 30BP (Cal BP 3,620–3,460). Guineu Cave is located in Font-Rubí (Barcelona, Spain) and the teeth studied come from the 4th and 3rd millineum BC, when the cave was used as a burial place^60^. Some dated human remains shows an age about 2,871–3,353 Cal BC.

El Mirador Cave is located on the southern side of the Sierra de Atapuerca (Burgos, Spain). The human assemblage where this tooth belongs to is a collective burial found in an about 14 m2 natural chamber located in the NE corner of the cave. Although there are some individuals in their original anatomical position, the superficial remains were mixed and disturbed by the actions of the clandestine excavators in the 1980s. Up to now, there are a minimum number of 22 individuals of different sexes and ages^61^. All of these human remains belong to the Chalcolithic period and have been dated to 4.760–4.200 years cal. BP.

The current Sudanese population comes from a sample from the North of Sudan (Jaali, Mahasi, Shaigi, Bedairi, Halfawi and Dongalawi groups)^62^.

#### Periodicity

To estimate the periodicity, naturally-broken teeth with exposed enamel from Sima de los Huesos (n = 10, MNI = 6), *H. antecessor* (n = 3) and Sima del Elefante-TE9 (n = 1), were selected. A complete list of the teeth can be seen in Table 1.

AT-6873 and AT-6874 are small fragments and thus we were unable to attribute tooth types. ATD6-104 and ATD6-92 correspond with a deciduous molar and a permanent molar respectively, although their position and side remains unknown.

## Methods

The dental development of the Atapuerca hominins was studied employing two complementary views. The first one deals with the evaluation of the absolute timing of crown formation using growth markings present in the enamel microstructure. The second one evaluates the relative formation time of all the teeth in a specific specimen compared to modern humans.

### Perikymata counts

Perikymata numbers were counted by using the environmental scanning electron microscope FEI Quanta 600, housed at the Human Evolution National Rearch Centre (CENIEH, Burgos, Spain) with the following settings: low vaccuum (60–80 Pa), voltage 15-16 kV, spot of 3.5–3.6 and intensity of 60 µA. The teeth were positioned obliquely to the electron beam in order to maximize the distance of their crown heights. Several images at maximum resolution (4096 × 3773 px) were collected at 50x–70x on their buccal aspects (lingual aspects in upper molars), attempting to capture as much dental surface as possible in order to be able to resolve the count of continuous perikymata when some part of the surface is worn or they are not clearly visible for any reason.

In order to increase the ease of counting the incremental lines, a highpass filter was used in all the pictures taken with the ESEM using software Darktable 2.0.1 and increasing the contrast boost up to 100%.

Crown heights in every tooth can be divided in ten equal segments, named deciles, which are numbered from 1 to 10, starting in the cuspal area or incisal edge and ending in the cervix. Perikymata were counted on every decile where they were recognizable (Fig. S4). These perikymata counts per population, decile and tooth were modeled running three-degree polynomial regressions to summarize three different rates of perikymata distribution, representing their 95% confidence limits (Table S7 and Fig. S5). To discern differences among perikymata distribution, we coded the overlap of the 95% confidence limits in every decile and tooth type between two species (Fig. 2).

In order to compare perikymata distributions, 95% confidence limits were taken into consideration. If an overlap exists in the confidence limits between two populations per tooth and decile, we consider it as not having differences. By contrast, if no overlap exists, we consider it as presenting differences. For instance, looking at upper M3 in Fig. S5, we considered perikymata distribution as not presenting differences from decile 1 to decile 5, because the confidence limits of both *H. sapiens* and Sima de los Huesos overlap. However, from decile 6 to decile 10 their confidence limits do not overlap, so they were considered as presenting a different rate of perikymata accumulation.

### Periodicity

As we were unable to obtain histological thin sections from the fossil specimens, we evaluated the periodicities of naturally fractured teeth with exposed enamel using the portable confocal scanning optical microscope (PCSOM)^63–65^. This confocal microscope is based on the Nipkow disc technique, which returns images from a very thin optical plane in a region immediately below the irregular fractured surface of the enamel (1–50 µm depending upon specimen characteristics).

In the sample of naturally fractured teeth, we selected one side of the exposed enamel surfaces per tooth focusing our analyses on the larger and more longitudinal inner exposed area that runs cuspal-cervically. Selected enamel surfaces were positioned slightly obliquely on the PCSOM stage to enable increased visual accessibility and detail of the enamel surfaces. In most cases, a cover slip was cut to fit the surface studied and immersion oil was used to increase contrast detail on the enamel surfaces. Images were taken by using a IEEE 1394 digital camera and the Infinity Analyze Release 6.5 software (Lumen*era* Corporation).

Three different objective lenses with varied magnifications were used: 5x, 10x and 20x. Each objective lens was used to mount a panorama of the fractured enamel surface. The field width of each objective lens corresponds to 20.44 mm (5x), 10.42 mm (10x), and 5.18 mm (20x), resulting in images with an XY spatial resolution of 13.20 µm/pixel (5x), 6.73 µm/pixel (10x), and 3.35 µm/pixel (20x). As the field of view is smaller than the complete fractured surface particularly when using the 10x and 20x lenses, several images were acquired to register the complete surface. In these instances, we imaged adjacent regions with a 25 to 30% of overlap so that these regions can be subsequently reconstructed without loosing detail. In every field view, 2 to 20 images were acquired by changing the Z projection in order to focus different and complementary regions of interest. The 5x lens allowed us to record the entire fractured surface in most cases, spanning from the outer enamel to the enamel-dentin junction (EDJ). The 10x and 20x allowed us to observe details of the enamel microstructure near the middle and outer enamel surface, but regions close to the EDJ were difficult to visualize. Only images obtained using the 20x objective lens were used to identify and to measure cross-striations, while striae of Retzius and perikymata were analyzed using the 10x and 20x lenses. From all specimens analyzed, a total of 1510 images were obtained.

All images were initially converted to 8-bit gray scale when imported to Fiji/ImageJ. As every panaroma is formed by several images, each of them formed by a stack of images with different focused areas, we first needed to align each set of images and to focus stack them subsequently. The alignment of the stack of images was done by using the ImageJ’s plug-in *Template Matching and Slice Alignment* with the following parameters: Matching mehotd = Normalized correlation coefficient, Subpixel registration checked and Search area (pixels around ROI) = 0. Focus stacking was done by using the ImageJ’s plug-in Extended Depth of Field^66^, with the following parameters: Sharpness estimator = Real wavelets, Wavelet basis: B-spline wavelets, Spline order = 5 and Number of scales = 11.

Once all the images of each panorama were generated, we imported them into the open-source software Inkscape (version 0.92) where we manually stitched them. Retzius lines were identified using the 10x panorama by ensuring their course reached the enamel surface and formed perikymata. The 20x panorama were used to measure the distance between adjacent Retzius lines following the course of the enamel prisms. These measurements were repeated several times. In those areas, we also measured several times the distance between 4 or 5 adjacent cross-striations (3 or 4 days respectively). Finally, we estimated the periodicities by using the mean value of the Retzius’ distances relative to the mean value of the cross striation’s distances. Daily secretion rates (µm/day) in the regions where the periodicities were estimated in each tooth were included in Table 1. These regions were coded and defined by Beynon *et al*. (1991)^67^.

### Statistical analysis and other software

The article was written using RMarkdown within RStudio 1.1.442 to ensure reproducibility^68,69^. The statistical analyses and manuscript layout required loading these set of R packages: captioner, to automatically number figure, table and text captions^70^; citr, to insert formatted Markdown citations from BibTex and Zotero^71^; ggbiplot, to represent biplots of the first two principal components after a PCA analysis^72^; ggplot2 and ggpubr, to graphically plot most of the figures of the article, including polar charts, boxplots and line plots^73,74^; knitr and kableExtra, to layout and export the manuscript in different formats, as.pdf,.html or.doc^75,76^; psych, to summarize data^77^; readODS, to import LibreOffice Calc databases into R^78^; reshape2, stringr, tidyverse, dplyr and plyr, to manipulate, transform and clean datasets^79–83^.

Some images were created or modified by using Inkscape 0.92 and GIMP 2.8. Zotero has been used as the reference management software, which has been connected to the R package citr and RStudio through the Zotero Better BibTex extension.

## Supplementary information

Supplementary information.

Supplementary information 2.
