# Supplementary material for: Short and long period growth markers of enamel formation distinguish European Pleistocene hominins
Source: Sci Rep. 2020 Mar 13;10:4665. doi: 10.1038/s41598-020-61659-y (PMC7069994; doi:10.1038/s41598-020-61659-y)
Supplement: Supplementary file 2 — Supplementary information 2. [file 41598_2020_61659_MOESM2_ESM.docx]

# Supplementary materials

## Supplementary texts

### Supplementary Text 1: New methodology to reconstruct the crown heights in slightly worn teeth

**Introduction**. Some of the fossil teeth included in this study are slightly worn, so that a reconstruction of the lost enamel is required. Crown heights of slightly worn fossil teeth were reconstructed in order to divide in ten deciles and count perikymata lines in each. For this purpose, we employed 116 unworn *H. sapiens* teeth (Table S8) to construct 7 regression equations which represent the cuspal morphology of each tooth type groups: incisors, canines, premolars and molars, both upper and lower. Lower molar regression was analyzed in a previous paper where the protocol of the methodology was detailed^17^. This teeth were obtained from the pool modern human teeth used to count perikymata (see Materials - Perikymata counts). With these regression equations, we validated the method by reconstructing artificially-worn unworn *H. sapiens* teeth and by comparing estimated versus real crown heights. Later, *H. antecessor* and Sima de los Huesos unworn teeth were also validated.

**Virtual scanning of the modern human unworn teeth**. Microtomographic scanning (µCT) of the modern human unworn teeth was performed at the CENIEH facilities (Burgos, Spain). Two scanners were employed: a Phoening v/tome/x s of GE Measurement system and a Scanco Medical AG Micro-Computed Tomography 80. The scan settings of the former were voltage 100 kV, intensity 100 µA and 0.02 cooper filter, while the scan settings of the latter were voltage 70 kV and intensity of of 114 µA. Resultant isometric voxel size ranges from 18-36 (isolated dental remains) to 75 (mandibles/maxillas) µm. µCT images were processed employing the software AMIRA 6.0.0 and AVIZO 8.0 (Visage Imaging, Inc.).

**Summary of the reconstruction method**. To reconstruct worn teeth, we employed the method and protocol described in detail in a previous paper^17^ for lower molars, but extended to other tooth types. This method is essentially based on the positioning of a regression equation that contains the average morphology of the cuspal area over a naturally worn tooth that preserves the dentin horn.

For *incisors* and *canines*, these reference slices were obtained following two steps. First, 10 equidistant landmarks are placed at the cervix of the buccal aspect and 10 more in the lingual aspect of the tooth, and an average oblique slice is calculated for those 20 landmarks. Second, by moving upwards and downwards the stack of images whose reference slice is the average oblique one, we identified three landmarks: the dentine horn in the canines or the highest point of the incisal edge, and the maximum enamel extensions both in the buccal and lingual aspects. The reference slices are those that are defined by the previous three landmarks. By default, the buccal side of the teeth are placed to the right.

The reference planes for *premolars* and *upper molars* were also obtained by following two steps. First, 50 equidistant landmarks are placed all along the cervix, and an average oblique slice is calculated. Second, the reference plane is that one that is perpendicular to the oblique slice and passes by the buccal and lingual dentine horns in the premolars, and protocone and paracone dentin horns in the upper molars. As incisors and canines, the buccal side of the teeth are placed to the right.

**Construction of the regressions**. Once all the reference slices were obtained, three new landmarks were placed on them (see Fig. 2 in^17^): 1) dentine horn, 2) point where the outline of the enamel is cut by a horizontal line traced that passes by the dentine horn and 3) point where the horizontal projection of the highest point of the cuspal enamel crosses the vertical line traced through the dentine horn. These three landmarks define a Cartesian system. In this Cartesian system, we focus on the enamel outline comprised between the newly created *X* and *Y* axis. This part of the enamel is divided en 100 equidistant segments, therefore defined by 101 points.

All these points were employed to create the regression equations with the training sample, each for each tooth type (Table S9). These regressions are fitted to a fourth polynomial model to keep their adjusted R-square above 0.9. The steps to reconstruct all tooth types are equivalent to those explained for lower molars^17^. All the raw files (.svg files and the converter) can be found in Supplementary File 1.

**Validation of the method**. We validated the new methodology by employing 116 unworn modern human teeth. We divided these teeth in 7 groups regarding its tooth type: upper and lower incisors, canines, premolars and molars (Table S8). To standardize the process of validation, every sample per tooth type was randomly subsampled in two groups using the function sample() in R. 4 teeth in every tooth type were artificially worn (these form the experimental sample). The remaining teeth were employed to construct the regression equations which were used to reconstruct the smaller subsample (these form the training sample). A comparison between the real and estimated crown heights was recorded.

The training sample sizes were shown to be largely enough to ensure that the inclusion or removal of a tooth would not significantly affect the curvature of the regression (Fig. S6).

The results are shown in Table S10. The mean percentage error in all tooth types but lower molars^17^ between both measurements is -0.55 ± 1.30% (0.98 ± 1.00% in absolute values). The maximum percentage error is present in the lower premolar Nº4 (-4.11%).

**Application of the method to the fossil teeth**. Replicating and validating the reconstruction methodology in fossil teeth is inconceivable due to a very low number or total absence of unworn tooth sample sizes. For this reason, we applied the *H. sapiens* regression equations to the fossil teeth, both from Sima de los Huesos and from *H. antecessor*. To validate the methodology in fossil hominins, we selected all the unworn fossil teeth from both sites. Lately, their cusps were artificially worn keeping the dentine horns present. Percentages of error between the real crown heights and the estimated ones were calculated. We considered a percentage error below 5% as acceptable, as this would mean that at least half of the first decile has been under/overestimated.

When reconstructing artificially-worn unworn fossil teeth (*H. antecessor*: n = 3, and Sima de los Huesos: n = 35) with regression equations based on *H. sapiens* teeth (Table S11 and Table S12), the mean percentage error between real and estimated crown heights is -1.01 ± 1.53% (1.42 ± 1.15% in absolute values). The highest difference (-4.50%) is present in AT-1752. Canines, premolars and molars, both upper and lower, behave similarly. No unworn fossil incisor exists, so the percentage error remains unknown. However, as all errors are below 5% in other tooth types and species, we assume this would also be the case in incisors.

Our cutoff in the percentage of difference is 5%, as this value would represent the lost of half of the first decile of the cuspal area. As shown, none of our estimations exceeds this value. Furthermore, our averages oscillate around zero, and those placed distantly are very scarce. This means that *H. sapiens* regressions can confidently be used to reconstruct *H. antecessor* and Sima de los Huesos slightly worn teeth.

### Supplementary Text 2: Minimum number of individuals when estimating periodicities in *H. antecessor* and Sima de los Huesos

The minimum number of individuals (MNI) in the *H. antecessor* sample where periodicity was evaluated is 3. However, only one tooth has a specimen attribution: ATD6-6 to hominin 1 (H1). The remaining teeth (ATD6-104 and ATD6-92) remains unattributed. Gran Dolina-TD6 unit can be divided in 3 subunits and 27 levels^84^. All these teeth were discovered in different Gran Dolina-TD6 levels, so they must necessarily belong to different specimens (Fig. S7).

The MNI in the Sima de los Huesos sample is 6. Five Sima de los Huesos teeth (AT-43, AT-64, AT-13, AT-2135 and AT-827) are attributed to four specific specimens (II, VII, XVIII and XIX). Two teeth from specimen II (AT-13 and AT-64) show the same periodicity value (8). Two other teeth from this sample were considered to belong to another two specimens. AT-1475 is an upper right canine with a 7-day periodicity. Specimens II and XIX lack this tooth type, but as they present 9 and 6 day-periodicity respectively, AT-1475 must be attributed to a different hominin. AT-1942 and AT-3192 are upper left and right canines respectively with 6-day periodicity both, so they could hypothetically belong to the XIX specimen, as it lacks both upper canines. However, their developmental stages and wear degrees are so different that they must be attributed to two different hominins (Fig. S8). By contrast, AT-6873 and AT-6874 are very small fragments which makes impossible to identify their tooth type and specimen attribution.

## Supplementary tables

| Upper | I1 | I2 | C | P3 | P4 | M1 | M2 | M3 | Total |
| --- | --- | --- | --- | --- | --- | --- | --- | --- | --- |
| TD6 | 0 | 2 | 2 | 4 | 3 | 3 | 1 | 0 | 15 |
| SH | 2 | 2 | 5 | 5 | 4 | 2 | 2 | 15 | 37 |
| SAP | 8 | 10 | 18 | 29 | 12 | 5 | 4 | 7 | 93 |
| Total | 10 | 14 | 25 | 38 | 19 | 10 | 7 | 22 | 145 |
| Lower | I1 | I2 | C | P3 | P4 | M1 | M2 | M3 | Total |
| TD6 | 0 | 0 | 1 | 1 | 2 | 1 | 1 | 1 | 7 |
| SH | 0 | 2 | 2 | 13 | 15 | 2 | 13 | 12 | 59 |
| SAP | 4 | 7 | 16 | 16 | 17 | 6 | 8 | 1 | 75 |
| Total | 4 | 9 | 19 | 30 | 34 | 9 | 22 | 14 | 141 |

Table S1: **Sample size of the teeth employed to count perikymata lines on the enamel surface**. Above: upper teeth; below: lower teeth.

|  | PC1 | PC2 | Accum |
| --- | --- | --- | --- |
| UI1 | 0.563 | 0.367 | 0.930 |
| UI2 | 0.658 | 0.229 | 0.887 |
| LI2 | 0.604 | 0.202 | 0.806 |
| UC | 0.692 | 0.160 | 0.852 |
| LC | 0.726 | 0.156 | 0.881 |
| UP3 | 0.721 | 0.143 | 0.864 |
| LP3 | 0.729 | 0.143 | 0.872 |
| UP4 | 0.560 | 0.221 | 0.781 |
| LP4 | 0.756 | 0.093 | 0.849 |
| UM1 | 0.786 | 0.127 | 0.913 |
| LM1 | 0.873 | 0.105 | 0.978 |
| UM2 | 0.731 | 0.184 | 0.915 |
| LM2 | 0.525 | 0.231 | 0.756 |
| UM3 | 0.671 | 0.242 | 0.913 |
| LM3 | 0.642 | 0.228 | 0.870 |

Table S2: **Values of percentage of variation explained by the first two principal components (PC1 and PC2) and its sum (Accum) per tooth type**. The principal component analysis was run on the perikymata number in the cervical deciles (from deciles 6 to 10, DC6-DC10). Tooth types are prefixed with U (Upper) and L (Lower).

| Tooth | Deciles | Comp.1 | Comp.2 | Comp.3 | Comp.4 | Comp.5 |
| --- | --- | --- | --- | --- | --- | --- |
| UI1 | DC6 | 0.13 | -0.26 | 0.61 | 0.65 | -0.34 |
| UI1 | DC7 | 0.05 | -0.46 | 0.47 | -0.73 | -0.17 |
| UI1 | DC8 | 0.04 | -0.60 | -0.03 | 0.18 | 0.78 |
| UI1 | DC9 | 0.25 | -0.56 | -0.63 | 0.07 | -0.48 |
| UI1 | DC10 | 0.96 | 0.23 | 0.05 | -0.08 | 0.15 |
| UI2 | DC6 | -0.10 | 0.05 | 0.11 | 0.94 | 0.30 |
| UI2 | DC7 | -0.20 | -0.14 | -0.88 | -0.05 | 0.41 |
| UI2 | DC8 | -0.20 | -0.83 | 0.35 | -0.13 | 0.35 |
| UI2 | DC9 | -0.32 | -0.40 | -0.24 | 0.26 | -0.78 |
| UI2 | DC10 | -0.90 | 0.35 | 0.19 | -0.16 | 0.08 |
| LI2 | DC6 | -0.07 | 0.85 | 0.51 | 0.05 | -0.06 |
| LI2 | DC7 | -0.14 | -0.18 | 0.24 | 0.89 | 0.31 |
| LI2 | DC8 | -0.48 | -0.41 | 0.67 | -0.37 | 0.11 |
| LI2 | DC9 | -0.55 | -0.03 | -0.15 | 0.22 | -0.79 |
| LI2 | DC10 | -0.67 | 0.27 | -0.46 | -0.10 | 0.51 |
| UC | DC6 | -0.49 | -0.41 | 0.12 | -0.09 | 0.76 |
| UC | DC7 | -0.47 | -0.25 | 0.28 | -0.57 | -0.56 |
| UC | DC8 | -0.55 | -0.12 | -0.16 | 0.75 | -0.31 |
| UC | DC9 | -0.38 | 0.45 | -0.73 | -0.33 | 0.08 |
| UC | DC10 | -0.30 | 0.74 | 0.59 | 0.08 | 0.13 |
| LC | DC6 | -0.38 | 0.71 | -0.26 | -0.26 | 0.48 |
| LC | DC7 | -0.46 | 0.34 | 0.03 | 0.03 | -0.82 |
| LC | DC8 | -0.47 | -0.05 | 0.78 | 0.30 | 0.28 |
| LC | DC9 | -0.53 | -0.57 | -0.13 | -0.61 | 0.03 |
| LC | DC10 | -0.39 | -0.24 | -0.56 | 0.68 | 0.12 |
| UP3 | DC6 | -0.05 | 0.29 | 0.19 | 0.54 | 0.76 |
| UP3 | DC7 | -0.16 | 0.52 | 0.34 | 0.46 | -0.62 |
| UP3 | DC8 | -0.27 | 0.62 | 0.20 | -0.69 | 0.19 |
| UP3 | DC9 | -0.61 | 0.16 | -0.76 | 0.16 | -0.02 |
| UP3 | DC10 | -0.73 | -0.49 | 0.48 | -0.01 | 0.03 |
| LP3 | DC6 | -0.25 | -0.19 | 0.39 | -0.59 | 0.64 |
| LP3 | DC7 | -0.33 | -0.32 | 0.45 | -0.24 | -0.73 |
| LP3 | DC8 | -0.38 | -0.62 | 0.03 | 0.64 | 0.25 |
| LP3 | DC9 | -0.53 | -0.09 | -0.77 | -0.33 | -0.08 |
| LP3 | DC10 | -0.63 | 0.69 | 0.24 | 0.26 | 0.05 |
| UP4 | DC6 | -0.20 | -0.12 | 0.44 | 0.75 | 0.43 |
| UP4 | DC7 | -0.31 | -0.60 | 0.53 | -0.24 | -0.44 |
| UP4 | DC8 | -0.38 | -0.33 | -0.20 | -0.45 | 0.71 |
| UP4 | DC9 | -0.51 | -0.23 | -0.66 | 0.39 | -0.32 |
| UP4 | DC10 | -0.67 | 0.68 | 0.23 | -0.16 | -0.08 |
| LP4 | DC6 | -0.25 | 0.13 | 0.75 | 0.40 | -0.45 |
| LP4 | DC7 | -0.26 | -0.20 | 0.56 | -0.51 | 0.57 |
| LP4 | DC8 | -0.42 | -0.68 | -0.18 | -0.28 | -0.51 |
| LP4 | DC9 | -0.47 | -0.29 | -0.19 | 0.67 | 0.46 |
| LP4 | DC10 | -0.69 | 0.64 | -0.25 | -0.24 | -0.06 |
| UM1 | DC6 | 0.03 | -0.59 | -0.11 | 0.17 | 0.78 |
| UM1 | DC7 | 0.15 | -0.63 | -0.30 | -0.57 | -0.40 |
| UM1 | DC8 | 0.00 | -0.08 | -0.69 | 0.65 | -0.30 |
| UM1 | DC9 | 0.14 | -0.46 | 0.65 | 0.46 | -0.37 |
| UM1 | DC10 | 0.98 | 0.18 | -0.04 | 0.02 | 0.09 |
| LM1 | DC6 | -0.04 | 0.71 | 0.37 | 0.52 | -0.29 |
| LM1 | DC7 | 0.14 | 0.52 | 0.24 | -0.79 | 0.14 |
| LM1 | DC8 | 0.50 | -0.35 | 0.75 | 0.12 | 0.23 |
| LM1 | DC9 | 0.74 | 0.01 | -0.30 | -0.06 | -0.60 |
| LM1 | DC10 | 0.42 | 0.31 | -0.40 | 0.28 | 0.70 |
| UM2 | DC6 | -0.06 | 0.73 | 0.49 | -0.33 | -0.33 |
| UM2 | DC7 | -0.09 | -0.31 | -0.29 | -0.87 | -0.24 |
| UM2 | DC8 | 0.05 | 0.11 | -0.47 | 0.34 | -0.81 |
| UM2 | DC9 | -0.01 | 0.60 | -0.67 | -0.10 | 0.43 |
| UM2 | DC10 | 0.99 | 0.02 | 0.03 | -0.12 | 0.00 |
| LM2 | DC6 | 0.16 | 0.71 | -0.05 | 0.39 | 0.56 |
| LM2 | DC7 | 0.19 | 0.43 | 0.02 | 0.31 | -0.82 |
| LM2 | DC8 | 0.29 | 0.43 | -0.02 | -0.85 | -0.03 |
| LM2 | DC9 | 0.55 | -0.24 | -0.79 | 0.09 | 0.02 |
| LM2 | DC10 | 0.74 | -0.26 | 0.61 | 0.11 | 0.09 |
| UM3 | DC6 | -0.08 | -0.18 | 0.49 | 0.38 | 0.76 |
| UM3 | DC7 | -0.12 | -0.18 | 0.82 | -0.16 | -0.50 |
| UM3 | DC8 | -0.30 | -0.39 | -0.09 | -0.80 | 0.33 |
| UM3 | DC9 | -0.63 | -0.54 | -0.27 | 0.43 | -0.24 |
| UM3 | DC10 | -0.70 | 0.70 | 0.08 | -0.06 | 0.06 |
| LM3 | DC6 | -0.23 | 0.58 | -0.46 | 0.55 | -0.29 |
| LM3 | DC7 | -0.37 | 0.19 | 0.42 | 0.38 | 0.71 |
| LM3 | DC8 | -0.81 | -0.09 | 0.29 | -0.23 | -0.44 |
| LM3 | DC9 | -0.37 | -0.06 | -0.69 | -0.42 | 0.46 |
| LM3 | DC10 | -0.14 | -0.78 | -0.22 | 0.57 | -0.03 |

Table S3: **Loading factors of cervical half deciles**. The loadings were classified by tooth type, species and principal component (Comp.1 to Comp.5). Tooth types are prefixed with U (Upper) and L (Lower).

| Site | Specimen | ID | Tooth | Position | Side |
| --- | --- | --- | --- | --- | --- |
| SH | I | AT-2027 | P3 | L | R |
| SH | I | AT-603 | P4 | L | L |
| SH | II | AT-142 | M2 | L | R |
| SH | II | AT-3179 | M2 | L | L |
| SH | III | AT-271 | M2 | L | R |
| SH | III | AT-273 | M2 | L | L |
| SH | III | AT-149 | P3 | L | R |
| SH | III | AT-47 | P3 | L | L |
| SH | IX | AT-169 | M2 | L | L |
| SH | IX | AT-277 | P4 | L | R |
| SH | VI | AT-1763 | P4 | L | L |
| SH | XI | AT-1761 | M2 | L | R |
| SH | XI | AT-557 | M2 | L | L |
| SH | XI | AT-148 | P3 | L | R |
| SH | XI | AT-168 | P4 | L | R |
| SH | XII | AT-171 | M3 | U | L |
| SH | XII | AT-194 | M3 | U | R |
| SH | XIV | AT-2272 | M2 | L | L |
| SH | XIV | AT-284 | M2 | L | R |
| SH | XIV | AT-1465 | P4 | L | L |
| SH | XIX | AT-816 | M3 | U | R |
| SH | XIX | AT-826 | M3 | U | L |
| SH | XVIII | AT-2165 | C | L | R |
| SH | XVIII | AT-410 | C | L | L |
| SH | XVIII | AT-2151 | C | U | L |
| SH | XVIII | AT-2207 | C | U | R |
| SH | XVIII | AT-1143 | I1 | U | L |
| SH | XVIII | AT-2395 | I1 | U | R |
| SH | XVIII | AT-2066 | I2 | L | L |
| SH | XVIII | AT-957 | I2 | L | R |
| SH | XVIII | AT-1124 | I2 | U | L |
| SH | XVIII | AT-2280 | I2 | U | R |
| SH | XVIII | AT-829 | M1 | L | L |
| SH | XVIII | AT-943 | M1 | L | R |
| SH | XVIII | AT-2071 | M1 | U | L |
| SH | XVIII | AT-2076 | M1 | U | R |
| SH | XVIII | AT-1752 | M2 | L | R |
| SH | XVIII | AT-941 | M2 | L | L |
| SH | XVIII | AT-2175 | M2 | U | R |
| SH | XVIII | AT-2179 | M2 | U | L |
| SH | XVIII | AT-2343 | P3 | L | R |
| SH | XVIII | AT-2767 | P3 | L | L |
| SH | XVIII | AT-2036 | P3 | U | L |
| SH | XVIII | AT-2399 | P3 | U | R |
| SH | XVIII | AT-2386 | P4 | L | R |
| SH | XVIII | AT-828 | P4 | L | L |
| SH | XVIII | AT-2070 | P4 | U | L |
| SH | XVIII | AT-2189 | P4 | U | R |
| SH | XX | AT-3890 | M2 | L | R |
| SH | XX | AT-946 | M2 | L | L |
| SH | XX | AT-3188 | P4 | L | R |
| SH | XX | AT-806 | P4 | L | L |
| SH | XXI | AT-1751 | P4 | L | L |
| SH | XXI | AT-562 | P4 | L | R |
| SH | XXII | AT-3180 | M3 | U | L |
| SH | XXII | AT-819 | M3 | U | R |
| SH | XXIII | AT-2387 | P4 | L | L |
| SH | XXVI | AT-30 | M3 | L | R |
| SH | ? | AT-1475 | C | U | R |
| SH | ? | AT-2388 | C | U | R |
| SH | ? | AT-44 | C | U | L |
| SH | ? | AT-143 | M3 | L | R |
| SH | ? | AT-1468 | M3 | L | R |
| SH | ? | AT-1945 | M3 | L | L |
| SH | ? | AT-1959 | M3 | L | R |
| SH | ? | AT-2273 | M3 | L | L |
| SH | ? | AT-2760 | M3 | L | L |
| SH | ? | AT-2777 | M3 | L | R |
| SH | ? | AT-3182 | M3 | L | R |
| SH | ? | AT-598 | M3 | L | L |
| SH | ? | AT-599 | M3 | L | R |
| SH | ? | AT-942 | M3 | L | R |
| SH | ? | AT-1471 | M3 | U | R |
| SH | ? | AT-274 | M3 | U | L |
| SH | ? | AT-3181 | M3 | U | L |
| SH | ? | AT-3183 | M3 | U | R |
| SH | ? | AT-5082 | M3 | U | R |
| SH | ? | AT-601 | M3 | U | R |
| SH | ? | AT-602 | M3 | U | L |
| SH | ? | AT-805 | M3 | U | R |
| SH | ? | AT-945 | M3 | U | R |
| SH | ? | AT-1466 | P3 | L | R |
| SH | ? | AT-1993 | P3 | L | L |
| SH | ? | AT-3243 | P3 | L | R |
| SH | ? | AT-4100 | P3 | L | L |
| SH | ? | AT-563 | P3 | L | L |
| SH | ? | AT-6728 | P3 | L | R |
| SH | ? | AT-807 | P3 | L | L |
| SH | ? | AT-41 | P3 | U | L |
| SH | ? | AT-5611 | P3 | U | L |
| SH | ? | AT-6181 | P3 | U | R |
| SH | ? | AT-221 | P4 | L | R |
| SH | ? | AT-2275 | P4 | L | L |
| SH | ? | AT-580 | P4 | L | L |
| SH | ? | AT-5510 | P4 | U | R |
| SH | ? | AT-746 | P4 | U | R |
| TD6 | H1 | ATD6-1 | C | L | L |
| TD6 | H1 | ATD6-13 | C | U | L |
| TD6 | H1 | ATD6-12 | M2 | U | R |
| TD6 | H1 | ATD6-3 | P3 | L | R |
| TD6 | H1 | ATD6-13 | P3 | U | L |
| TD6 | H1 | ATD6-7 | P3 | U | R |
| TD6 | H1 | ATD6-125 | P4 | L | R |
| TD6 | H1 | ATD6-4 | P4 | L | R |
| TD6 | H1 | ATD6-8 | P4 | U | R |
| TD6 | H1 | ATD6-9 | P4 | U | L |
| TD6 | H10 | ATD6-113 | M2 | L | L |
| TD6 | H10 | ATD6-113 | M3 | L | L |
| TD6 | H3 | ATD6-69 | C | U | R |
| TD6 | H3 | ATD6-69 | I2 | U | R |
| TD6 | H3 | ATD6-69 | M1 | U | L |
| TD6 | H3 | ATD6-69 | M1 | U | R |
| TD6 | H3 | ATD6-69 | P3 | U | L |
| TD6 | H3 | ATD6-69 | P3 | U | R |
| TD6 | H3 | ATD6-69 | P4 | U | R |
| TD6 | H5 | ATD6-103 | M1 | U | R |
| TD6 | H6 | ATD6-312 | I2 | U | L |
| TD6 | ? | ATD6-94 | M1 | L | R |

Table S4: ***H. antecessor* (TD6) and Sima de los Huesos (SH) teeth where perikymata number were counted**.

| ID | Specimen | Tooth | Position | Side | % lost |
| --- | --- | --- | --- | --- | --- |
| ATD6-94 |  | M1 | L | R | 3.554576 |
| ATD6-103 | H5 | M1 | U | R | 9.458041 |
| ATD6-69 | H3 | M1 | U | L | 11.493136 |
| ATD6-69 | H3 | M1 | U | R | 11.811958 |
| ATD6-69 | H3 | P3 | U | L | 3.602251 |
| ATD6-69 | H3 | P3 | U | R | 3.175500 |
| ATD6-113 | H10 | M2 | L | L | 10.418863 |
| ATD6-113 | H10 | M3 | L | L | 4.825695 |
| ATD6-1 | H1 | C | L | L | 9.691956 |
| ATD6-12 | H1 | M2 | U | R | 11.229309 |
| ATD6-125 | H1 | P4 | L | R | 15.960843 |
| ATD6-13 | H1 | C | U | L | 6.402456 |
| ATD6-13 | H1 | P3 | U | L | 5.441259 |
| ATD6-3 | H1 | P3 | L | R | 10.953242 |
| ATD6-4 | H1 | P4 | L | R | 3.240185 |
| ATD6-7 | H1 | P3 | U | R | 2.015481 |
| ATD6-8 | H1 | P4 | U | R | 5.176132 |
| ATD6-9 | H1 | P4 | U | L | 5.447011 |

Table S5: **Percentages of lost enamel in slightly worn *H. antecessor* (TD6) teeth**. They were calculated by employing the regression equations.

| ID | Specimen | Tooth | Position | Side | % lost |
| --- | --- | --- | --- | --- | --- |
| AT-1124 | XVIII | I2 | U | L | 0.6329559 |
| AT-1143 | XVIII | I1 | U | L | 3.0875126 |
| AT-142 | II | M2 | L | R | 4.6372727 |
| AT-1465 | XIV | P4 | L | L | 4.8101828 |
| AT-1466 |  | P3 | L | R | 1.7803333 |
| AT-1475 |  | C | U | R | 4.7463545 |
| AT-148 | XI | P3 | L | R | 4.5956888 |
| AT-149 | III | P3 | L | R | 5.9388468 |
| AT-168 | XI | P4 | L | R | 1.6907362 |
| AT-169 | IX | M2 | L | L | 7.7415865 |
| AT-171 | XII | M3 | U | L | 7.6186624 |
| AT-1751 | XXI | P4 | L | L | 11.3361242 |
| AT-1761 | XI | M2 | L | R | 6.8702593 |
| AT-1763 | VI | P4 | L | L | 3.6461071 |
| AT-194 | XII | M3 | U | R | 1.5382846 |
| AT-1945 |  | M3 | L | L | 1.0959204 |
| AT-1993 |  | P3 | L | L | 3.1120414 |
| AT-2027 | I | P3 | L | R | 2.1574432 |
| AT-2066 | XVIII | I2 | L | L | 3.8604108 |
| AT-2071 | XVIII | M1 | U | L | 9.3386875 |
| AT-2076 | XVIII | M1 | U | R | 11.2892752 |
| AT-221 |  | P4 | L | R | 4.2975633 |
| AT-2272 | XIV | M2 | L | L | 9.7373931 |
| AT-2280 | XVIII | I2 | U | R | 1.3903656 |
| AT-2387 | XXIII | P4 | L | L | 4.1984984 |
| AT-2388 |  | C | U | R | 3.8177046 |
| AT-2395 | XVIII | I1 | U | R | 3.5164182 |
| AT-271 | III | M2 | L | R | 5.4275557 |
| AT-273 | III | M2 | L | L | 14.3483636 |
| AT-277 | IX | P4 | L | R | 3.5596478 |
| AT-284 | XIV | M2 | L | R | 6.8250711 |
| AT-30 | XXVI | M3 | L | R | 2.2713375 |
| AT-3179 | II | M2 | L | L | 3.2238474 |
| AT-3180 | XXII | M3 | U | L | 13.1699171 |
| AT-3183 |  | M3 | U | R | 3.0818540 |
| AT-3243 |  | P3 | L | R | 3.0626230 |
| AT-3890 | XX | M2 | L | R | 3.1340869 |
| AT-41 |  | P3 | U | L | 1.6676202 |
| AT-4100 |  | P3 | L | L | 4.0838413 |
| AT-44 |  | C | U | L | 5.2789949 |
| AT-47 | III | P3 | L | L | 10.5375135 |
| AT-5082 |  | M3 | U | R | 8.7821559 |
| AT-557 | XI | M2 | L | L | 9.3695586 |
| AT-562 | XXI | P4 | L | R | 8.8109834 |
| AT-563 |  | P3 | L | L | 6.0975011 |
| AT-580 |  | P4 | L | L | 5.3974812 |
| AT-603 | I | P4 | L | L | 2.0577498 |
| AT-6181 |  | P3 | U | R | 6.1158921 |
| AT-6728 |  | P3 | L | R | 1.3827496 |
| AT-807 |  | P3 | L | L | 1.2648910 |
| AT-819 | XXII | M3 | U | R | 17.9586950 |
| AT-826 | XIX | M3 | U | L | 3.1719671 |
| AT-829 | XVIII | M1 | L | L | 5.0665316 |
| AT-943 | XVIII | M1 | L | R | 4.9118250 |
| AT-957 | XVIII | I2 | L | R | 3.4514638 |

Table S6: **Percentages of lost enamel in slightly worn Sima de los Huesos (SH) teeth**. They were calculated by employing the regression equations.

| Tooth | Species | r2_1 | r2_2 | r2_3 | Intercept | Coef1 | Coef2 | Coef3 |
| --- | --- | --- | --- | --- | --- | --- | --- | --- |
| UI1 | SAP | 0.61 | 0.68 | 0.68 | 11.76 | -1.00 | 0.29 | 0.00 |
| UI1 | SH | 0.72 | 0.72 | 0.78 | 32.64 | -14.40 | 2.65 | -0.13 |
| LI1 | SAP | 0.63 | 0.72 | 0.72 | 15.41 | -3.34 | 0.75 | -0.03 |
| UI2 | SAP | 0.78 | 0.84 | 0.85 | 3.98 | 2.51 | -0.40 | 0.04 |
| UI2 | SH | 0.78 | 0.78 | 0.87 | 19.33 | -10.65 | 2.48 | -0.14 |
| UI2 | TD6 | 0.57 | 0.67 | 0.69 | 5.34 | 0.82 | 0.52 | -0.05 |
| LI2 | SAP | 0.69 | 0.79 | 0.79 | 12.01 | -1.69 | 0.41 | -0.01 |
| LI2 | SH | 0.74 | 0.82 | 0.85 | 13.41 | -6.75 | 1.83 | -0.11 |
| UC | SAP | 0.63 | 0.66 | 0.66 | 11.60 | -1.73 | 0.62 | -0.03 |
| UC | SH | 0.66 | 0.69 | 0.69 | 8.34 | -1.15 | 0.45 | -0.02 |
| UC | TD6 | 0.63 | 0.73 | 0.74 | 13.33 | -4.24 | 0.92 | -0.04 |
| LC | SAP | 0.64 | 0.68 | 0.68 | 14.47 | -3.02 | 0.86 | -0.04 |
| LC | SH | 0.78 | 0.83 | 0.83 | 8.72 | 1.53 | -0.21 | 0.02 |
| LC | TD6 | 0.87 | 0.96 | 0.96 | 8.83 | -2.08 | 0.42 | -0.01 |
| UP3 | SAP | 0.69 | 0.80 | 0.80 | 10.14 | -2.54 | 0.54 | -0.01 |
| UP3 | SH | 0.77 | 0.78 | 0.79 | 5.69 | -0.51 | 0.29 | -0.01 |
| UP3 | TD6 | 0.68 | 0.69 | 0.70 | 11.89 | -4.76 | 1.08 | -0.05 |
| LP3 | SAP | 0.71 | 0.79 | 0.79 | 8.50 | 0.14 | 0.02 | 0.01 |
| LP3 | SH | 0.64 | 0.68 | 0.68 | 6.38 | -0.44 | 0.17 | 0.00 |
| LP3 | TD6 | 0.73 | 0.89 | 1.00 | 398.11 | -166.07 | 22.79 | -1.00 |
| UP4 | SAP | 0.62 | 0.70 | 0.70 | 10.69 | -3.09 | 0.73 | -0.03 |
| UP4 | SH | 0.80 | 0.83 | 0.86 | 8.06 | -3.12 | 0.83 | -0.04 |
| UP4 | TD6 | 0.78 | 0.84 | 0.84 | 5.24 | 0.25 | 0.04 | 0.01 |
| LP4 | SAP | 0.67 | 0.80 | 0.80 | 9.72 | -1.42 | 0.27 | 0.00 |
| LP4 | SH | 0.76 | 0.79 | 0.79 | 5.99 | -0.81 | 0.28 | -0.01 |
| LP4 | TD6 | 0.66 | 0.70 | 0.70 | 14.39 | -2.33 | 0.36 | -0.01 |
| UM1 | SAP | 0.52 | 0.77 | 0.85 | 3.41 | 4.75 | -1.30 | 0.11 |
| UM1 | SH | 0.43 | 0.47 | 0.53 | 833.50 | -306.50 | 37.50 | -1.50 |
| UM1 | TD6 | 0.64 | 0.68 | 0.69 | 10.19 | -2.65 | 0.53 | -0.02 |
| LM1 | SAP | 0.51 | 0.57 | 0.58 | 9.57 | -2.59 | 0.64 | -0.03 |
| LM1 | SH | 0.67 | 0.67 | 0.85 | 142.80 | -68.11 | 10.55 | -0.50 |
| LM1 | TD6 | 0.66 | 0.90 | 0.90 | -5.31 | 6.77 | -0.69 | 0.02 |
| UM2 | SAP | 0.64 | 0.80 | 0.82 | 5.04 | 1.81 | -0.50 | 0.05 |
| UM2 | SH | 0.81 | 0.93 | 0.94 | 8.32 | -3.26 | 0.72 | -0.03 |
| UM2 | TD6 | 0.70 | 0.70 | 0.83 | 62.80 | -40.17 | 8.75 | -0.58 |
| LM2 | SAP | 0.79 | 0.87 | 0.87 | 7.56 | -1.24 | 0.32 | -0.01 |
| LM2 | SH | 0.76 | 0.80 | 0.81 | 7.10 | -2.50 | 0.59 | -0.03 |
| LM2 | TD6 | 0.79 | 0.86 | 0.95 | -58.43 | 27.82 | -3.73 | 0.17 |
| UM3 | SAP | 0.63 | 0.84 | 0.86 | 5.57 | 1.51 | -0.55 | 0.06 |
| UM3 | SH | 0.66 | 0.82 | 0.83 | 5.07 | -0.05 | -0.07 | 0.02 |
| LM3 | SAP | 0.87 | 0.88 | 0.90 | 7.77 | -3.51 | 1.09 | -0.06 |
| LM3 | SH | 0.75 | 0.84 | 0.84 | 5.72 | -0.95 | 0.24 | 0.00 |
| LM3 | TD6 | 0.62 | 0.88 | 0.89 | -9.10 | 3.90 | 0.16 | -0.03 |

Table S7: **Coefficients of determination of linear models (r2_1), second, and third degree polynomial models (r2_2, r2_3), and intercepts and coefficients of the third degree polynomial models**. TD6: *H. antecessor*; SH: Sima de los Huesos hominins; SAP: *Homo sapiens*.

| Position | Tooth | Training | Experimental | Total |
| --- | --- | --- | --- | --- |
| Upper | Incisors | 11 | 4 | 15 |
| Lower | Incisors | 7 | 4 | 11 |
| Upper | Canines | 13 | 4 | 17 |
| Lower | Canines | 12 | 4 | 16 |
| Upper | Premolars | 19 | 4 | 23 |
| Lower | Premolars | 19 | 4 | 23 |
| Upper | Molars | 7 | 4 | 11 |
|  | Total | 88 | 28 | 116 |

Table S8: **Sample sizes of unworn modern human teeth employed in the process of validation of the cuspal area reconstruction methodology**. Number of teeth used to create the regression equations (*Training*) and those virtually worn to validate the methodology (*Experimental*) are shown.

|  | Upper I | Lower I | Upper C | Lower C | Upper P | Lower P | Upper M |
| --- | --- | --- | --- | --- | --- | --- | --- |
| Intercept | 9.29e+01 | 7.51e+01 | 9.14e+01 | 7.75e+01 | 8.59e+01 | 8.93e+01 | 9.40e+01 |
| X | 7.38e-01 | 2.00e+00 | 7.91e-01 | 1.47e+00 | 5.81e-01 | 9.74e-01 | 8.75e-01 |
| X^2 | -3.09e-02 | -5.20e-02 | -2.44e-02 | -3.28e-02 | -2.39e-03 | -3.33e-02 | 3.68e-02 |
| X^3 | 2.44e-04 | 4.23e-04 | 1.16e-04 | 1.01e-04 | -2.20e-04 | 2.23e-04 | 2.79e-04 |
| X^4 | -1.02e-06 | -1.78e-06 | -4.20e-07 | 3.35e-08 | 1.01e-06 | -7.59e-07 | 9.17e-07 |
| Adj. R-sq | 0.975 | 0.941 | 0.975 | 0.9 | 0.951 | 0.961 | 0.984 |

Table S9: **Fourth degree regression equations**. Incisors (I), canines (C), premolars (P) and molars (M), and their adjusted R-squared (Adj. R.sq).

| Tooth | UI | LI | UC | LC | UP | LP | UM |
| --- | --- | --- | --- | --- | --- | --- | --- |
| Nº1 (REL) | -0.8947443 | 0.0185667 | 0.5667199 | -0.6218091 | 0.7294438 | -1.0028702 | 1.4139997 |
| Nº1 (ABS) | 0.8947443 | 0.0185667 | 0.5667199 | 0.6218091 | 0.7294438 | 1.0028702 | 1.4139997 |
| Nº2 (REL) | -0.2728165 | -1.1832052 | -0.6310992 | -2.5101678 | 1.0837515 | -0.0932132 | 0.7151705 |
| Nº2 (ABS) | 0.2728165 | 1.1832052 | 0.6310992 | 2.5101678 | 1.0837515 | 0.0932132 | 0.7151705 |
| Nº3 (REL) | -0.4084354 | 1.2018293 | -1.1217680 | -3.7289179 | -0.5118542 | -1.8147474 | 0.2555011 |
| Nº3 (ABS) | 0.4084354 | 1.2018293 | 1.1217680 | 3.7289179 | 0.5118542 | 1.8147474 | 0.2555011 |
| Nº4 (REL) | -0.1595208 | -0.2724401 | -0.1056664 | -0.1197359 | -0.9061107 | -4.1130676 | -0.9234077 |
| Nº4 (ABS) | 0.1595208 | 0.2724401 | 0.1056664 | 0.1197359 | 0.9061107 | 4.1130676 | 0.9234077 |
| Mean (REL) | 0.0000000 | 0.0092833 | 0.2833599 | 0.0000000 | 0.9065977 | 0.0000000 | 1.0645851 |
| sd (REL) | 0.7637609 | 0.9661425 | 0.6103944 | 2.1114907 | 0.2045596 | 0.8223695 | 0.4034692 |
| Mean (ABS) | 0.0007306 | 0.2843481 | -0.4835395 | 1.2362931 | 0.1792780 | 0.8063134 | 1.0645851 |
| sd (ABS) | 0.8708833 | 0.7738134 | 1.4687642 | 0.8718778 | 0.9296021 | 0.5551985 | 0.4941468 |

Table S10: **Percentage errors between real and estimated crown heights in artificially-worn unworn *H. sapiens* teeth**. Data for the four artificially-worn teeth of the experimental sample (Nº1, Nº2, Nº3, Nº4), as well as means (Mean) and standard deviations (sd) are shown. Both relative (REL) and absolute (ABS) error values for upper incisors (UI), lower incisors (LI), upper canines (UC), lower canines (LC), upper premolars (UP), lower premolars (LP) and upper molars (UM) are displayed.

| Site | ID | Tooth | Position | Side | REL | ABS |
| --- | --- | --- | --- | --- | --- | --- |
| SH | AT-2165 | C | L | R | -0.8035019 | 0.8035019 |
| SH | AT-410 | C | L | L | -0.7387550 | 0.7387550 |
| SH | AT-2207 | C | U | R | -0.3817738 | 0.3817738 |
| SH | AT-2151 | C | U | L | 0.0591450 | 0.0591450 |
| SH | AT-2343 | P3 | L | R | 0.1591847 | 0.1591847 |
| SH | AT-2767 | P3 | L | L | 0.9919317 | 0.9919317 |
| SH | AT-2399 | P3 | U | R | -0.4916731 | 0.4916731 |
| SH | AT-2036 | P3 | U | L | 0.4617023 | 0.4617023 |
| SH | AT-5611 | P3 | U | L | 1.8014848 | 1.8014848 |
| SH | AT-828 | P4 | L | L | -0.7579564 | 0.7579564 |
| SH | AT-2386 | P4 | L | R | 1.0247214 | 1.0247214 |
| SH | AT-746 | P4 | U | R | 0.1383928 | 0.1383928 |
| SH | AT-2070 | P4 | U | L | 0.4415613 | 0.4415613 |
| SH | AT-2189 | P4 | U | R | 0.9343170 | 0.9343170 |
| SH | AT-1752 | M2 | L | R | -4.4989318 | 4.4989318 |
| SH | AT-941 | M2 | L | L | -2.0991686 | 2.0991686 |
| SH | AT-2175 | M2 | U | R | -1.9625968 | 1.9625968 |
| SH | AT-2179 | M2 | U | L | -0.9231412 | 0.9231412 |
| SH | AT-2273 | M3 | L | L | -3.9319480 | 3.9319480 |
| SH | AT-2777 | M3 | L | R | -3.3884931 | 3.3884931 |
| SH | AT-1959 | M3 | L | R | -3.2736791 | 3.2736791 |
| SH | AT-942 | M3 | L | R | -2.6227022 | 2.6227022 |
| SH | AT-2760 | M3 | L | L | -2.4016743 | 2.4016743 |
| SH | AT-3182 | M3 | L | R | -2.1165967 | 2.1165967 |
| SH | AT-1468 | M3 | L | R | -2.0722596 | 2.0722596 |
| SH | AT-143 | M3 | L | R | -1.8418660 | 1.8418660 |
| SH | AT-599 | M3 | L | R | -1.5641448 | 1.5641448 |
| SH | AT-598 | M3 | L | L | 0.2615519 | 0.2615519 |
| SH | AT-602 | M3 | U | L | -2.3199083 | 2.3199083 |
| SH | AT-1471 | M3 | U | R | -0.9675802 | 0.9675802 |
| SH | AT-601 | M3 | U | R | -0.6969798 | 0.6969798 |
| SH | AT-945 | M3 | U | R | -0.6860544 | 0.6860544 |
| SH | AT-805 | M3 | U | R | -0.4722150 | 0.4722150 |
| SH | AT-274 | M3 | U | L | 0.0000000 | 0.0000000 |
| SH | AT-3181 | M3 | U | L | 1.0036336 | 1.0036336 |
| TD6 | ATD6-112 | M1 | L | R | 0.5000000 | 0.5000000 |
| TD6 | ATD6-69 | M2 | U | L | -2.4000000 | 2.4000000 |
| TD6 | ATD6-5 | M3 | L | R | -2.9000000 | 2.9000000 |

Table S11: **Percentage errors between real and estimated crown heights in artificially-worn fossil teeth from *H. antecessor* (TD6) and Sima de los Huesos (SH)**. Both relative (REL) and absolute (ABS) error values are displayed.

|  | Min. | 1st Qu. | Median | Mean | 3rd Qu. | Max. |
| --- | --- | --- | --- | --- | --- | --- |
| ABS | 0.0 | 0.49 | 0.98 | 1.42 | 2.11 | 4.5 |
| REL | -4.5 | -2.11 | -0.75 | -1.01 | 0.15 | 1.8 |

Table S12: **Numerical summary of the percentage errors between real and estimated crown heights in artificially-worn fossil teeth from *H. antecessor* (TD6) and Sima de los Huesos (SH).** Both relative (REL) and absolute (ABS) error values are displayed.

## Supplementary figures


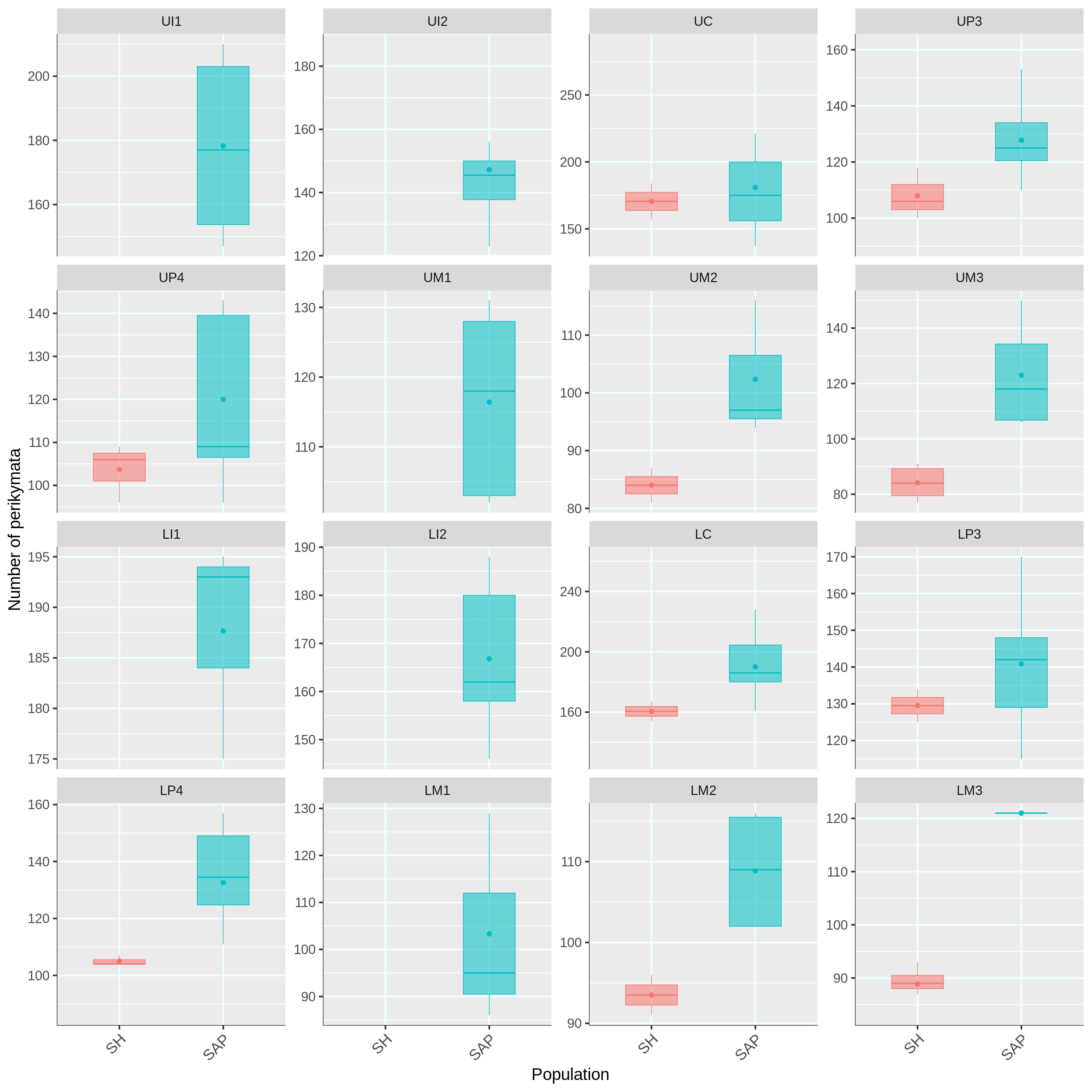


Fig. S1: **Boxplots representing total perikymata counts in the entire crown**. The dots in the boxplots represent the mean. Sima de los Huesos (SH), and *H. sapiens* (SAP).


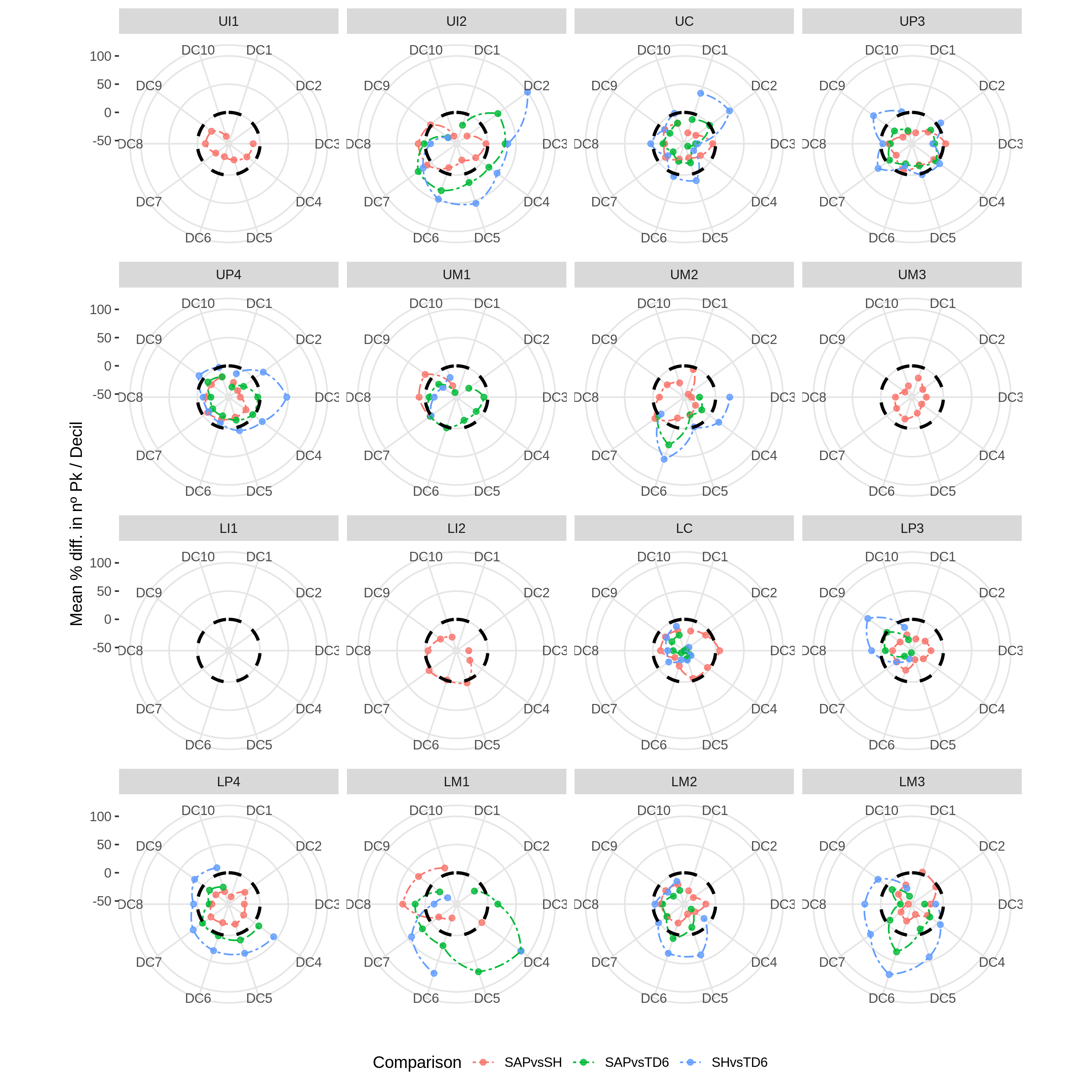


Fig. S2: **Polar charts representing the percentage of difference in number of perikymata per decile and tooth**. In all cases, the first species in the comparison is represented by the black dashed line which is zero. SAPvsSH: *H. sapiens* vs. Sima de los Huesos; SAPvsTD6: *H. sapiens* vs. *H. antecessor*; SHvsTD6: Sima de los Huesos vs. *H. antecessor*.


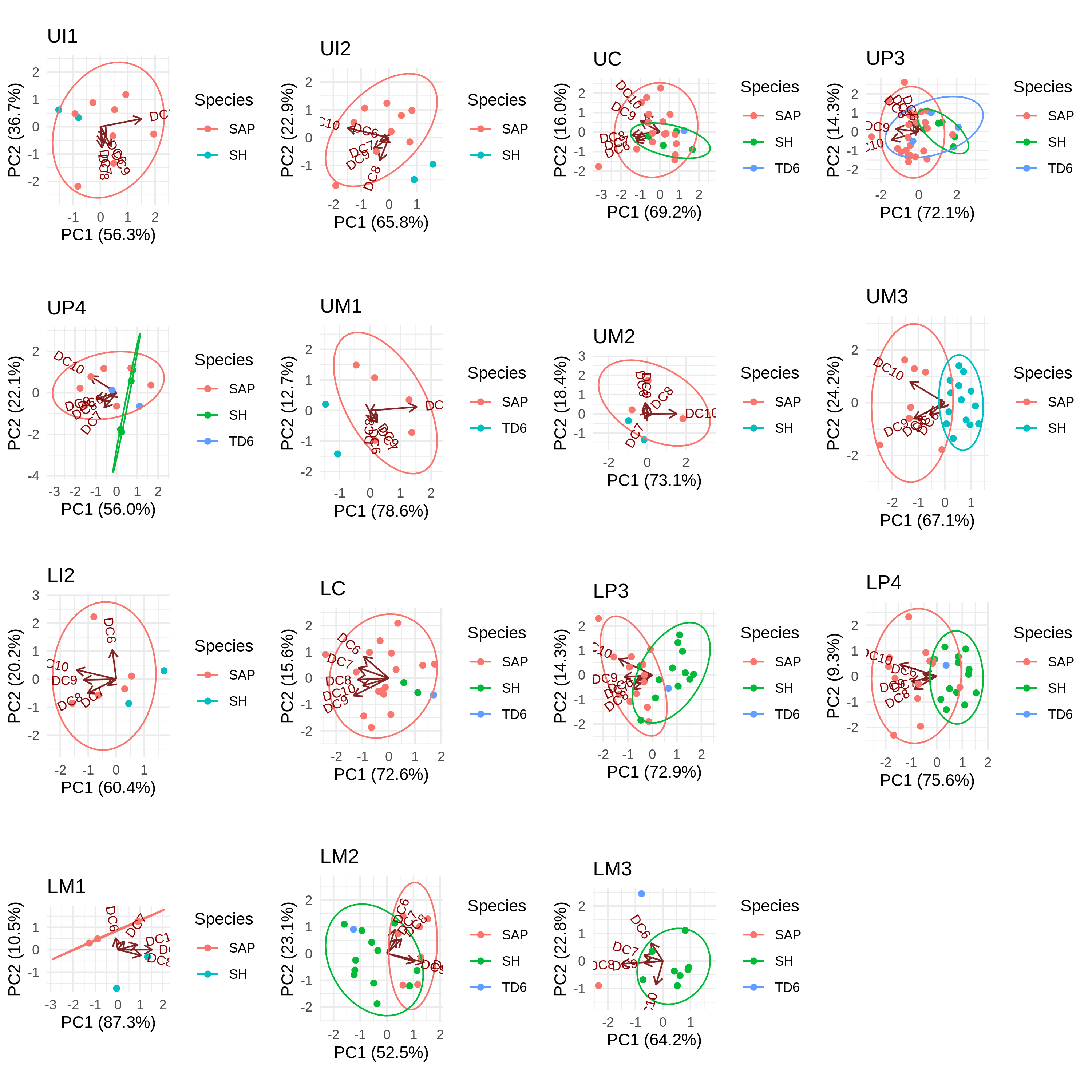


Fig. S3: **Biplots representing the first two principal components (PC1 and PC2) of the number of perikymata in the cervical deciles**. Ellipses represent the equiprobability of 0.9. Tooth types are prefixed with U (Upper) and L (Lower). TD6: *H. antecessor*; SH: Sima de los Huesos; SAP: *H. sapiens*.


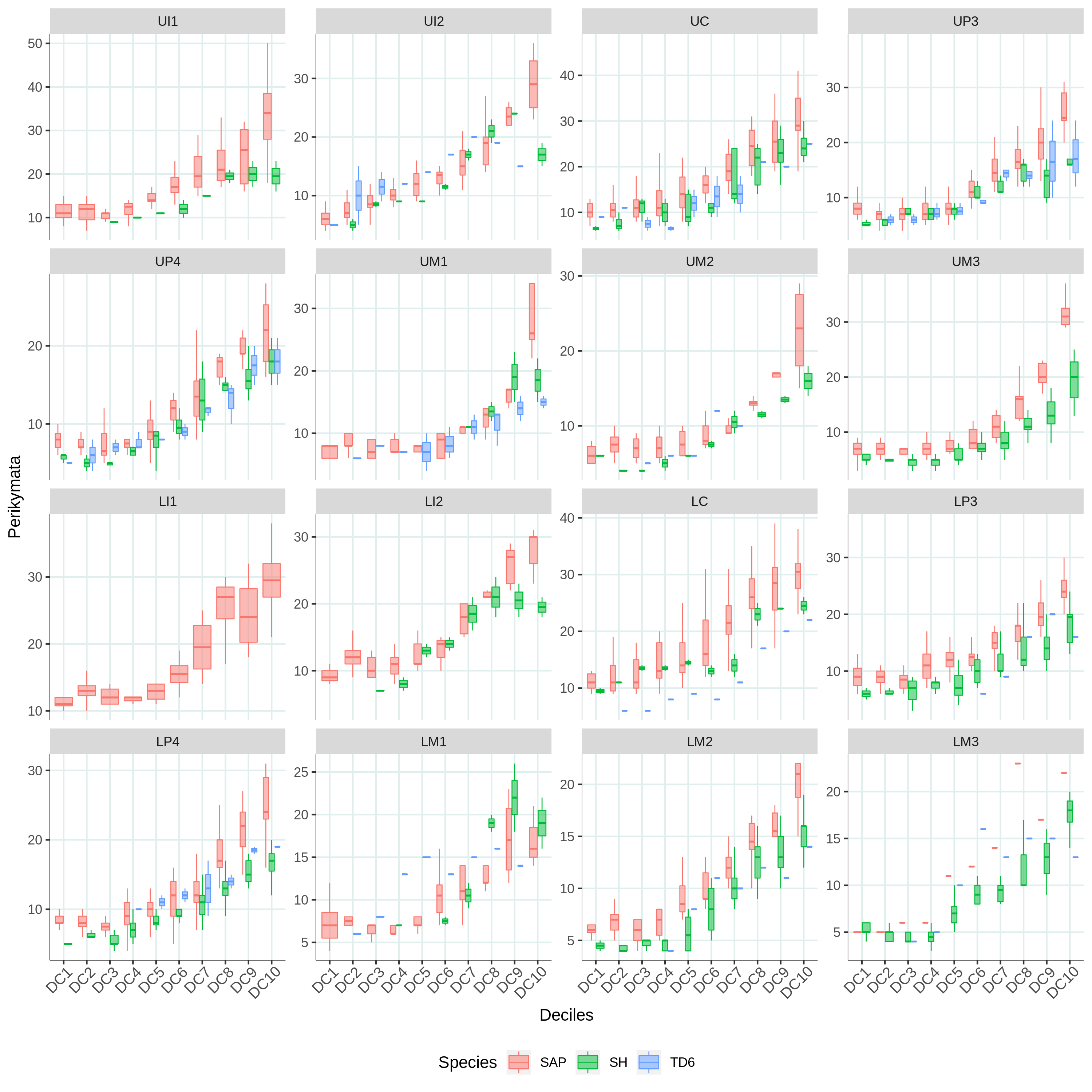


Fig. S4: **Boxplots representing perikymata counts per decile in all teeth**. *H. antecessor* (TD6), Sima de los Huesos (SH), and *H. sapiens* (SAP).


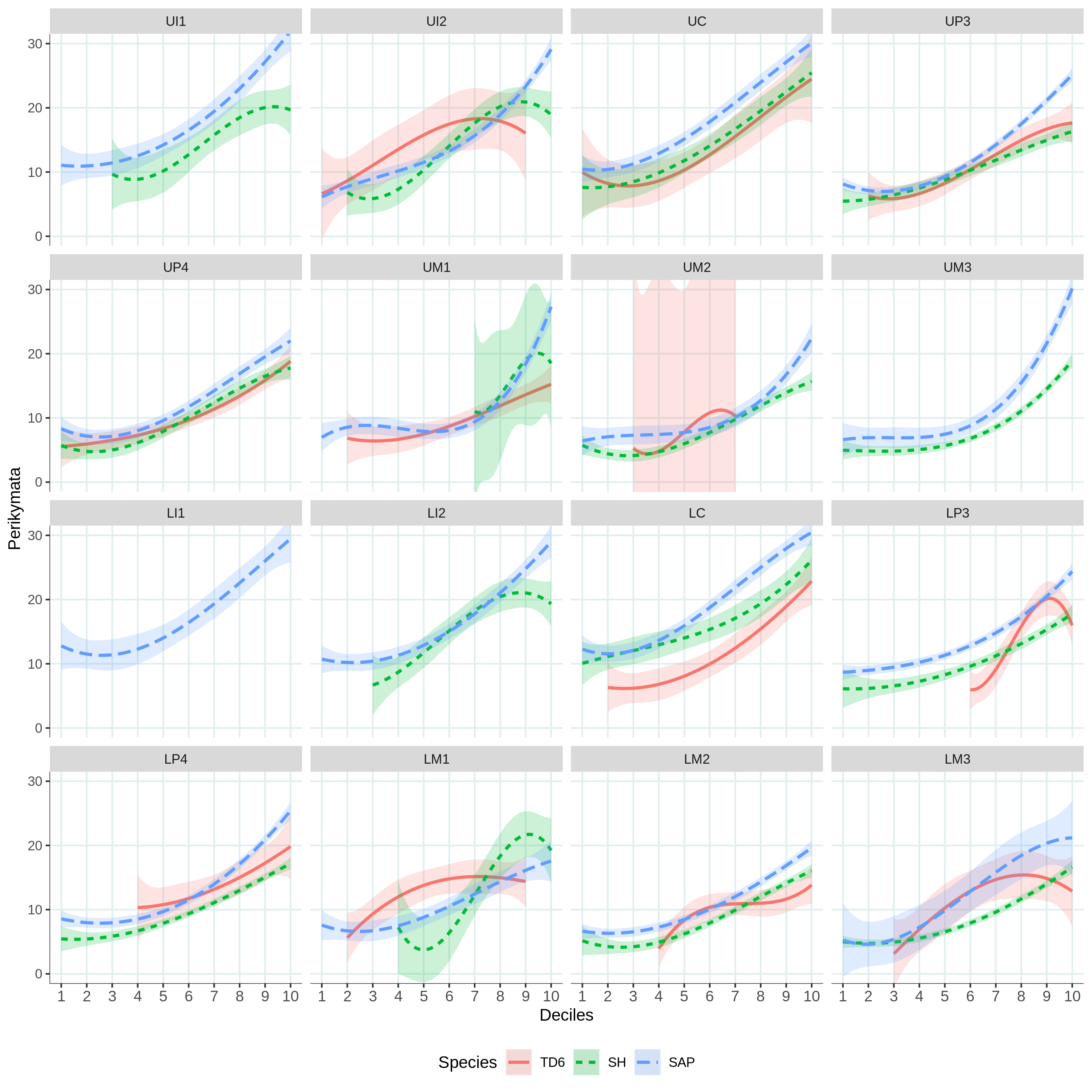


Fig. S5: **Three-degree polynomial regressions of the perikymata distribution in the deciles of every population and tooth**. 95% confidence limits are also represented. *H. antecessor* (TD6), Sima de los Huesos (SH), and *H. sapiens* (SAP). U = Upper; L = Lower.


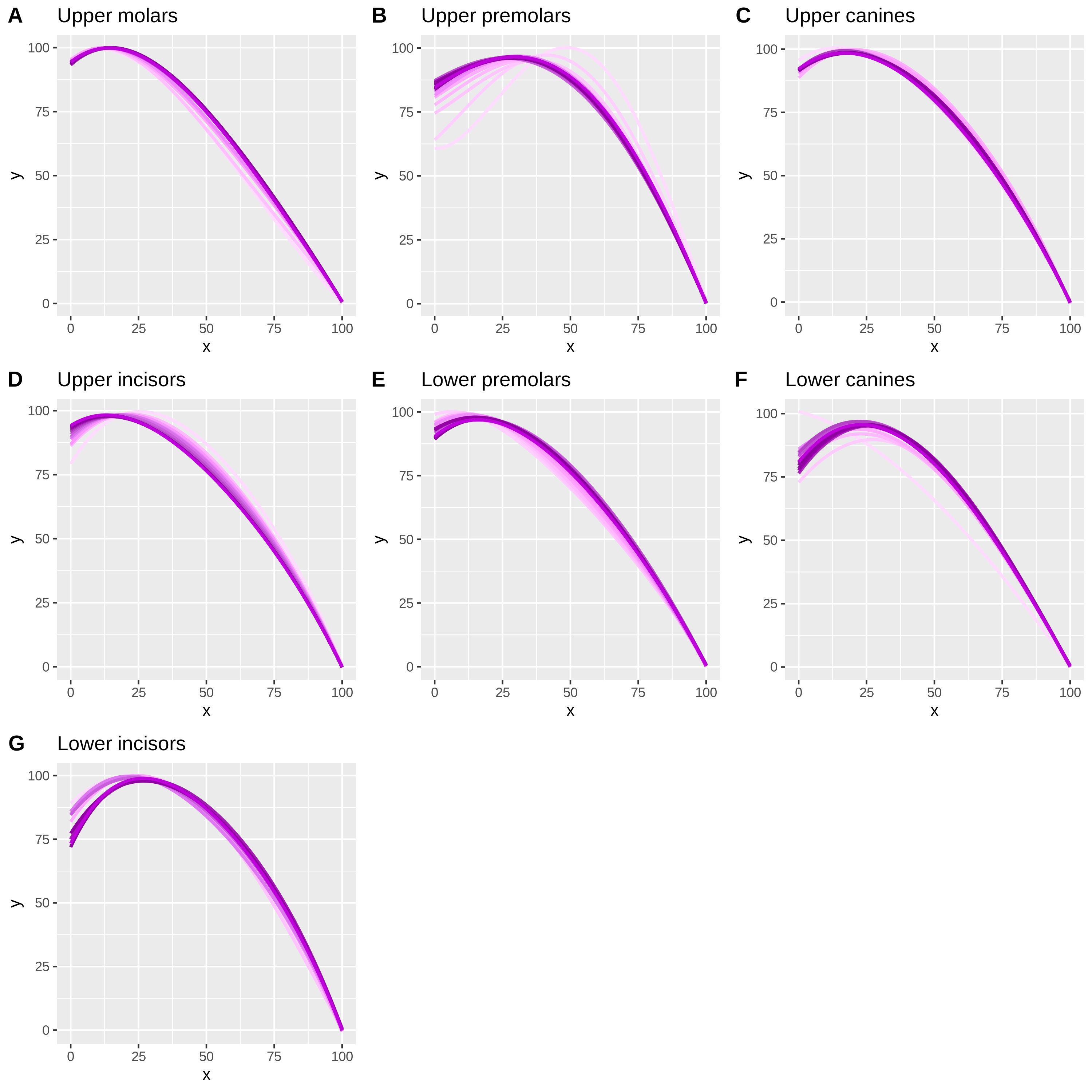


Fig. S6: **Accumulative regression equations per tooth type**. The darker the purple color is, the more number of teeth are included in the construction of the regression equation.


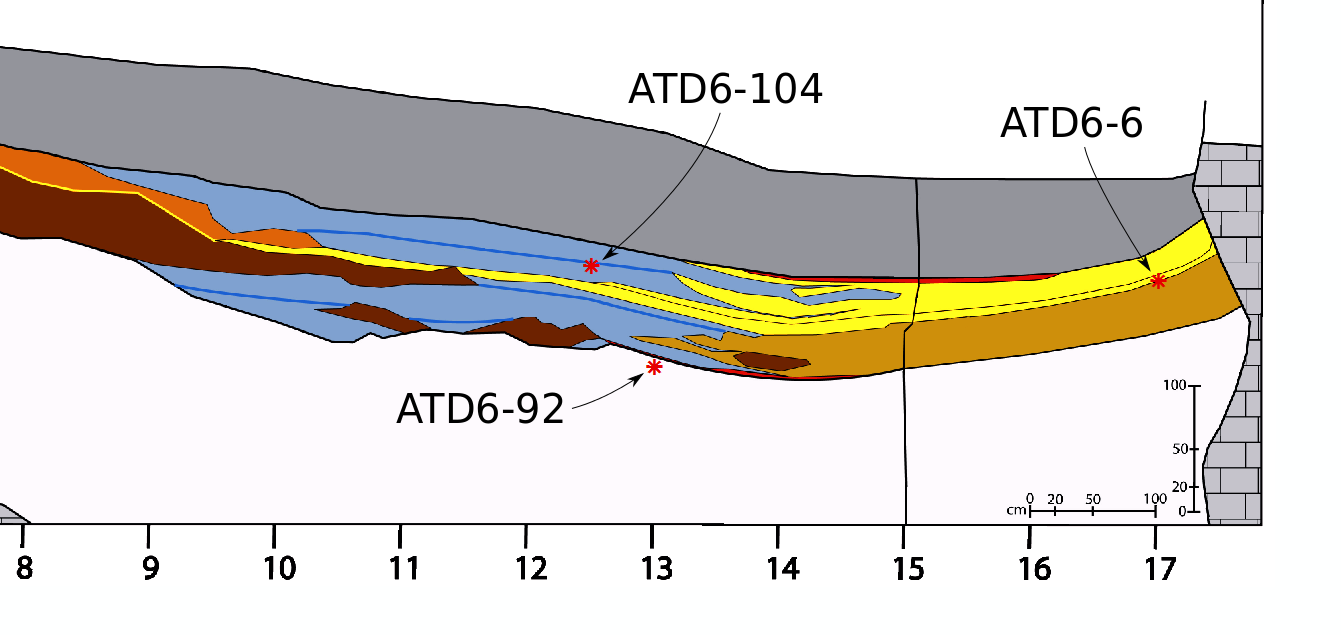


Fig. S7: **Location of the three *H. antecessor* teeth in the stratigraphy of the TD6 level**.


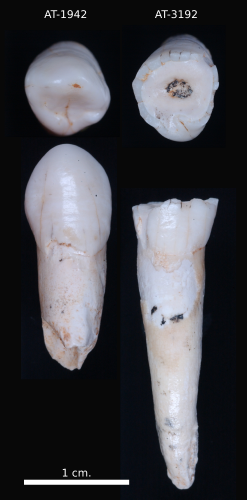


Fig. S8: **Comparison of two upper canines from SH (AT-1942 and AT-3192)**. Clear differences in the wear stages are shown.

## Supplementary files

Supplementary File 1: **Zip folder containing the raw files in .svg of the regression equations and the converter**.
